# Dynamic transcriptome analysis reveals signatures of paradoxical effect of vemurafenib on human dermal fibroblasts

**DOI:** 10.1186/s12964-021-00801-3

**Published:** 2021-12-20

**Authors:** Eyleen Corrales, Ella Levit-Zerdoun, Patrick Metzger, Silke Kowar, Manching Ku, Tilman Brummer, Melanie Boerries

**Affiliations:** 1 Institute of Molecular Medicine and Cell Research (IMMZ), University of Freiburg, Stefan-Meier-Str. 17, 79104 Freiburg, Germany; 2 Institute of Medical Bioinformatics and Systems Medicine (IBSM), Medical Center-University of Freiburg, Faculty of Medicine, University of Freiburg, Breisacherstr. 153, 79110 Freiburg, Germany; 3 Faculty of Biology, University of Freiburg, Schänzlestr. 1, 79104 Freiburg, Germany; 4 German Cancer Research Center (DKFZ), Im Neuenheimer Feld 280, 69120 Heidelberg, Germany; 5 German Cancer Consortium (DKTK), Freiburg, Germany; 6 Department of Pediatrics and Adolescent Medicine, Division of Pediatric Hematology and Oncology, Medical Center-University of Freiburg, Faculty of Medicine, University of Freiburg, Mathildenstr. 1, 79106 Freiburg, Germany; 7 Centre for Biological Signalling Studies (BIOSS), University of Freiburg, Schänzlestr. 18, 79104 Freiburg, Germany

**Keywords:** Vemurafenib, PLX8394, Fibroblast, Melanoma, Transcriptome, Chromatin

## Abstract

**Background:**

Vemurafenib (PLX4032) is one of the most frequently used treatments for late-stage melanoma patients with the BRAF^*V600E*^ mutation; however, acquired resistance to the drug poses as a major challenge. It remains to be determined whether off-target effects of vemurafenib on normal stroma components could reshape the tumor microenvironment in a way that contributes to cancer progression and drug resistance.

**Methods:**

By using temporally-resolved RNA- and ATAC-seq, we studied the early molecular changes induced by vemurafenib in human dermal fibroblast (HDF), a main stromal component in melanoma and other tumors with high prevalence of BRAF^*V600*^ mutations.

**Results:**

Transcriptomics analyses revealed a stepwise up-regulation of proliferation signatures, together with a down-regulation of autophagy and proteolytic processes. The gene expression changes in HDF strongly correlated in an inverse way with those in BRAF^*V600E*^ mutant malignant melanoma (MaMel) cell lines, consistent with the observation of a paradoxical effect of vemurafenib, leading to hyperphosphorylation of MEK1/2 and ERK1/2. The transcriptional changes in HDF were not strongly determined by alterations in chromatin accessibility; rather, an already permissive chromatin landscape seemed to facilitate the early accessibility to MAPK/ERK-regulated transcription factor binding sites. Combinatorial treatment with the MEK inhibitor trametinib did not preclude the paradoxical activation of MAPK/ERK signaling in HDF. When administered together, vemurafenib partially compensated for the reduction of cell viability and proliferation induced by trametinib. These paradoxical changes were restrained by using the third generation BRAF inhibitor PLX8394, a so-called paradox breaker compound. However, the advantageous effects on HDF during combination therapies were also lost.

**Conclusions:**

Vemurafenib induces paradoxical changes in HDF, enabled by a permissive chromatin landscape. These changes might provide an advantage during combination therapies, by compensating for the toxicity induced in stromal cells by less specific MAPK/ERK inhibitors. Our results highlight the relevance of evaluating the effects of the drugs on non-transformed stromal components, carefully considering the implications of their administration either as mono- or combination therapies.

**Video Abstract**

**Supplementary Information:**

The online version contains supplementary material available at 10.1186/s12964-021-00801-3.

## Background

The substitution of valine by glutamic acid at codon 600 (V600E) is the most frequently occurring mutation in the B-rapidly accelerated fibrosarcoma (BRAF) gene [1]. It confers RAS-independent constitutive BRAF activity, leading to hyperactivation of the downstream mitogen-activated protein kinase/extracellular signal-regulated kinase (MAPK/ERK) pathway, which in turn promotes proliferation, de-differentiation, and cell survival [1, 2]. The V600E substitution is present in about 40–60% of all melanomas and recapitulation experiments in zebrafish and mouse models confirm this mutations as a driver event of melanoma tumorigenesis [3]. In addition, other tumors such as colorectal adenocarcinoma, papillary thyroid cancer, and lung adenocarcinoma also exhibit high prevalence of the mutation [4]. Altogether, this has encouraged the development of therapies that specifically target the mutated BRAF.

In 2011, the FDA approved the use of PLX4032, also known as vemurafenib (short for *V*600*E* *mu*tated B*RAF* *in*h*ib*itor), as treatment of late-stage melanoma [5]. This drug blocks the activation of the mitogen-activated protein kinase kinase (MAPK/ERK kinase; MEK) through an ATP-competitive inhibition of the kinase domain of BRAF with the mutations V600E and, to a minor extent, V600K [6], providing a median progression-free survival (PFS) of about seven months [7–9].

Although this, as well as other available protein kinase inhibitors, are considerably specific in comparison to cytotoxic chemotherapeutics, they also exert off-target effects. Vemurafenib shows a similar potency for BRAF^*V600E*^ (IC50 = 31 nM) and CRAF (IC50 = 48 nM). Moreover, although it shows a higher selectivity for the mutated kinase, it also inhibits the BRAF^*WT*^ (IC50 = 100 nM) and several non-RAF kinases in vitro [6].

In BRAF^*WT*^ melanoma cells, vemurafenib (or its tool compound PLX4720) can stimulate the kinase activity of BRAF dimers leading to a paradoxical increase of MEK activation [10–12]. Therefore, the therapy has been reserved for the treatment of patients with mutant BRAF^*V600E/K*^ alleles. However, patients undergoing vemurafenib treatment can still experience off-target effects of the drug on their BRAF^*WT*^ stroma cells.

Given the recognized role of the microenvironment as a key modulator of the tumor progression, a detailed assessment of the molecular effects of this or any other drugs on the stromal cells is desirable; however, this frequently remains overlooked. It has been shown that inhibition with vemurafenib can induce secretome changes in drug-stressed tumor cells which support the development of resistance [13, 14]. These tumor-promoting secretomes can act by promoting the establishment of supportive microenvironments. However, it remains to be elucidated whether the drugs additionally have a direct impact on different stroma components and reprogram them in a way that could further promote tumor progression.

Here, in a systems biology approach, we address the detailed dynamic effects of vemurafenib on the transcriptome and chromatin architecture of fibroblasts as a major component of the stroma in melanoma [15, 16], wherein patients could profit from the use of inhibitors targeting BRAF^*V600*^ mutations. We predict that the implementation of unbiased approaches to gain a better understanding of the major molecular changes, and signaling pathways possibly affected in stromal cells, could guide to better treatment decisions that improve the tumor response while minimizing the undesirable side effects on patients.

## Methods

### Cell culture

Primary cell cultures were established from human dermal fibroblast (HDF), isolated from juvenile foreskin of anonymous healthy donors who underwent circumcision through the Department of Dermatology of the University Medical Center, Freiburg. The non-immortalized HDF cultures were used within the first eight passages. All the specimens were collected with informed consent, according to the regulations of the University Medical Center, Freiburg. Patient-derived BRAF^*V600E*^-positive malignant melanoma cell lines included in this study (MaMel21, MaMel63a, MaMel19, and MaMel86b) were provided by Prof. Dr. Stefan Eichmüller. The primary cultures were established at the German Cancer Research Center (DKFZ), Heidelberg, from skin biopsies from female donors at stage IV of the disease. BRAF^*WT*^ melanoma cell lines were either purchased from Rockland Immunochemicals (WM3438), or kindly provided by Dr. Meenhard Herlyn, Philadelphia (SBcl2).

The cell lines were maintained at 37 °C in a humidified atmosphere of 5% CO_2_ using RPMI-1640 medium supplemented with 10% FCS, 1% L-glutamine, and 1% penicillin–streptomycin. Pharmacological inhibition experiments on HDF were performed using 2 µM of PLX4032 or PLX8394, 5 nM trametinib, a combination of the inhibitors, or an analog volume of vehicle (0.2% of DMSO) as control, for the indicated time for every experiment.

### RNA sequencing and preprocessing

Changes in HDF after vemurafenib treatment were evaluated at the transcriptome level using temporally-resolved RNA-seq. To this end, HDF seeded at a density of 53,000 cells/cm^2^ were stimulated for 4 h, 8 h or 18 h with 2 µM of vemurafenib or an analog volume of vehicle. Each condition was assessed in duplicates within every experiment, and two independent experiments were carried out, for a total of four sequenced replicates per treatment condition.

The cell pellets were collected by scrapping, and total RNA was isolated using the Universal RNA Purification Kit (Roboklon), following the manufacturer’s instructions. TruSeq library preparation and paired-end sequencing (2 × 100 bp) on NovaSeq 6000 instrument (Illumina) was carried out at the Genomics and Proteomics Core Facility of the DKFZ, Heidelberg. Between 59.4 and 106.2 million total reads were obtained for each sample.

Sequence quality was evaluated with FastQC [17], and adapter trimming and filtering of low-quality reads was performed with Trimmomatic [18]. A trailing approach was used to remove low quality bases with a Phred score lower than 20. All reads with less than 35 bp were discarded. The remaining reads were then mapped to the Ensembl GRCh37 reference genome with the STAR aligner [19], using the GRCh37.75 annotation GTF file to determine the number of fragments overlapping each Ensembl gene. Non-uniquely mapped reads were discarded. In addition, genes that failed to achieve a minimum of one count-per-million (CPM) in at least two of the samples were filtered out as unexpressed.

Compositional and size differences between the libraries were normalized using the Trimmed Mean of M-values (TMM) method [20], as implemented in the Bioconductor limma R package [21–23]. Raw counts were then transformed to log_2_ CPM with the associated precision weights, using the voom method [24].

For this study, RNA-seq was also performed for BRAF^*WT*^ melanoma cell lines, SBcl2 and WM3438, after 18 h and 24 h of stimulation with 2 µM of vemurafenib or an analog volume of vehicle. Each condition was assessed in duplicates. We additionally integrated our own dataset from BRAF^*V600E*^ positive melanoma cell lines, MaMel21 and MaMel63a, treated for 24 h with an identical concentration of the inhibitor. A matrix with the normalized counts for each cell line can be found in the Additional file 1: Table S1–Additional file 3: Table S3.

### Differentially expressed gene (DEG) analysis

Prior to DEG analysis, all Ensembl identifiers with no official gene symbol were filtered out, retaining a total of 12,865 genes in HDF, and 14,578 genes in MaMel cell lines for all downstream analysis (12,396 genes shared between both cell types). To detect the DEGs between time-matched vemurafenib-treated cells and control groups, empirical Bayes-moderated t-statistics were calculated with limma [25]. A paired design was used to account for baseline differences between the two experimental replicates for HDF, whereas an unpaired design was implemented for the analysis of the dataset from the other cell lines. Gene expression changes were considered significant at a Benjamini–Hochberg false discovery rate (FDR) adjusted *p*-value (*q*-value) cutoff of 0.05, unless otherwise stated. A list of DEGs can be found in the Additional file 4: Table S4–Additional file 6: Table S6.

### Soft clustering of time-course expression profiles

To identify dynamic gene expression changes in HDF after vemurafenib treatment, soft clustering analysis was performed based on fuzzy c-means algorithm, as implemented on the Mfuzz R package [26]. This method estimates for every gene a probability of membership in each of the defined clusters, generating a soft partitioning of the data based on the temporal expression profiles of the genes.

To this end, log_2_ fold changes (FC) were computed between time-matched vemurafenib-treated HDF and control groups, and clustering was performed in Euclidian space for the top 30% of genes (n = 3860) with the highest additive FC over time. The Dmin function was used to define the optimal number of clusters (c = 3) through visual inspection for a plateau in the minimum centroid distance within a given range of cluster numbers (c = 2–10). A minimum membership score of 0.6 was required to assign a gene to a particular cluster.

### Functional annotation analysis

To identify processes that are likely to be affected by vemurafenib treatment, functional over-representation analysis of gene ontology biological processes (GO-bp) was done with the R package clusterProfiler [27] for the DEGs and subsets of soft clustered genes. For the annotation of DEGs, up- and down-regulated genes were considered independently. Over-representation test was performed for functional categories with a set size of 10–500 genes, and these were considered to be significantly over-represented with a q-value cutoff of 0.1, unless otherwise stated. Visualization of over-represented terms was done in Cytoscape 3.7.2. The enrichmentMap plugin was used to construct a similarity-based network of the enriched biological processes. GO terms were represented as nodes and interconnecting edges were drawn based on a combined similarity coefficient (Jaccard + Overlap score > 0.4). Annotation of clusters of enriched terms was done with the AutoAnnotate plugin, using the Markov Cluster Algorithm (MCL).

Gene-set enrichment analysis was done with the R package GAGE [28], to test for enrichment of signature genes of interests. Paired testing was used to account for baseline differences between biological replicates. Functional categories were considered significant with a q-value cutoff of 0.05.

### Assay for transposase-accessible chromatin (ATAC) sequencing and preprocessing

Chromatin accessibility profiles from HDF after vemurafenib treatment were reconstructed using temporally-resolved ATAC-seq. The experimental conditions used for the ATAC-seq were identical to those for RNA-seq, with each condition assessed within two independent replicates. The cells were collected by trypsinization, and counted with the CASY system (Innovatis, Roche Applied Science).

From each condition, 50,000 cells were taken to prepare ATAC-seq reactions according to the original protocol from Buenrostro et al. [29]. A total of 12 PCR cycles were used to amplify the libraries after transposition. Double size selection and cleanup of the amplified libraries was done with AMPure XP beads (Beckman Coulter), using 1:0.5 and 1:1.5 sample-to-reagent ratios. The final quality of the libraries was assessed using the 2200 TapeStation system (Agilent Technologies). Paired-end sequencing (2 × 100 bp) of ATAC-seq libraries was performed on the NovaSeq 6000 instrument (Illumina) by the Genomics and Proteomics Core Facility of the DKFZ, Heidelberg. Between 145.9 and 227.2 million total reads were obtained for each sample.

Sequence quality was evaluated with FastQC [17]. Adapter trimming and quality filtering was performed with Trimmomatic [18], using a trailing approach to remove bases with a Phred score below 20. All reads with less than 25 bp were discarded, and those remaining were mapped to the Ensembl GRCh37 reference genome with the Bowtie 2 aligner [30], allowing a maximum insert size of 2000.

Alignments with a MAPQ score bellow 30, or unproperly paired, were discarded with SAMtools [31]. Duplicates were removed using Picard MarkDuplicates [32], and reads mapping to the mitochondrial chromosome were also discarded. Between 53.0 and 94.5 million of mapped reads were kept after filtering. Retained reads were shifted with deepTools alignmentSieve [33], to adjust the 5’ end of the reads to match the center of the Tn5 transposase binding site.

### Peak calling and annotation

Peak calling for ATAC-seq samples was performed on libraries down-sampled to ~ 53 million reads with Picard DownsampleSam. Peak regions were collectively called from the replicates with Genrich [34], using the ATAC-seq mode, and peak calling thresholds of AUC > 200 and *q*-value < 0.01. ENCODE blacklisted regions were excluded from the called peaks [35]. The genomic location of the peaks was annotated with the R package ChIPseeker [36], using the Bioconductor TxDb.Hsapiens.UCSC.hg19.knownGene database. Peaks were annotated as promoter peaks, when located within a region of ± 3 kb around the transcriptional start site (TSS). A complete list of the identified ATAC-seq peaks from each experimental condition can be found in the Additional file 7: Table S7, with the first columns in the standard narrowPeak format, and additional columns providing the annotated genomic location of the peaks.

### Differentially accessible region (DAR) analysis

Differential accessibility analysis within the identified ATAC-seq peak regions was performed according to the workflow described by Reske et al. [37]. Briefly, the R package soGGi [38] was used to define consensus regions among all the peaks from the different experimental conditions. ATAC-seq reads within these regions were counted, and loess-based normalization offsets were computed with the csaw R package [39, 40]. Low signal-abundance regions (log_2_ CPM < -3), or those located within or close to non-expressed genes were excluded from further analysis. The remaining 23,134 regions were subjected to differential accessibility analysis between time-matched vemurafenib-treated HDF and control groups, using edgeR to compute empirical Bayes estimation and perform quasi-likelihood F-test [41, 42]. Before FDR adjustment, proximal regions within a distance < 500 bp apart were merged, restraining the maximal merged window size to 5 kb. For merged regions, the most significant one was used as a statistical representation of the window. A total of 21,800 accessibility regions were kept after merging. Chromatin changes were considered significant with a q-value cutoff of 0.1, unless otherwise stated.

To determine whether differential chromatin accessibility could predict the gene expression changes detected through RNA-seq, unweighted receiver operating characteristic (ROC) and precision-recall (PR) curves were computed, using the PRROC R package [43]. To this aim, the most significant promoter DARs were selected for each expressed gene, and the -log_10_ transformed q-value was used to predict the boolean DEG status, binarized according to an RNA-seq q-value threshold of 0.05 (i.e., 1: DEG, 0: non-significant DEG).

### Transcription factor (TF) binding motif analysis

Identification of enriched TF binding motifs in sets of promoter peaks was done with monaLisa R package [44]. To this aim, promoter peaks with a minimum absolute log_2_ FC of 0.4 were grouped into bins of 100 peaks based on their fold changes calculated through differential accessibility analysis as detailed above. Enrichment of motifs from the JASPAR 2018 database was estimated within the bins by comparison to all other regions.

De novo motif discovery within specific ATAC-seq peak regions of interest was performed with the Bioconductor package rGADEM using masking of low-complexity sequences [45]. The identified motifs were compared to the JASPAR database using the Bioconductor package MotIV [46–48] to identify those with potential biological relevance.

### Flow cytometry

For assessment of the proliferative state of the cells, intracellular staining of Ki67 proliferation marker was performed. To this end, the cells were harvested and resuspended in a dilution of Fixable Viability Dye eFluor 780 (1:1000; eBioscience), to label and exclude dead cells from the analysis. After 10 min of labeling at room temperature, the unbound dye was washed out with PBS and then fixation and permeabilization of the cells was done with 70% ice cold ethanol, added dropwise. To ensure appropriate permeabilization, the samples were stored at – 20 °C for at least 4 h. Blocking was done for 30 min at room temperature with a blocking solution (3% FCS on PBS). Staining was then performed for 1 h at room temperature with anti-Ki67-eFluor 450 monoclonal antibody (1:100; SolA15; eBioscience) diluted in blocking solution. The cells were then rinsed with the blocking solution and subjected to flow cytometry acquisition.

To evaluate cellular viability, Annexin V and propidium iodide (PI) staining was performed. For this purpose, the medium containing all non-attached dead cells was collected. The cells were then harvested and the cell suspensions were pooled back together with the corresponding previously collected cell culture medium. The cells were resuspended in an Annexin V-FITC dilution (1:25; BD Bioscience) on 1 × Binding Buffer and incubated for 10 min at room temperature. After washing of the unbound Annexin V, the cells were resuspended in a PI dilution (1:50; BD Bioscience) and subjected to flow cytometry analysis.

Flow cytometry acquisition from all experiments was performed on a BD LSR-II analyzer (BD Biosciences), using the DB FACSDiva software. A total of 10,000 events were acquired for every assessed condition. Data analysis was done using FlowJo 10.3 software.

### MTT assay

The concentration of vemurafenib required to cause 50% growth inhibition (IC50) on MaMel cells was determined by MTT assay. To this end, 10,000 cells were plated in triplicates in 96-well plates, and allowed to attach overnight. The cells were then treated with different concentrations of vemurafenib, ranging from 15 pM to 20 µM. After 48 h of inhibitor treatment, the cell viability was assessed by adding 200 µl of 200 mg/ml MTT (Calbiochem), incubated for 2.5 h at 37 °C. The MTT solution was then removed, and 200 µl of DMSO were added to each well. Absorbance was measured at 565 nm.

### Real-time cell analysis (RTCA)

To monitor cell migration (chemotaxis) and invasion in real-time, the xCELLigence System (ACEA Biosciences) was used. In this system, electrical impedance changes are measured through a set of gold microelectrodes fused to the bottom of Boyden chambers (CIM Plate). The impedance, expressed as Cell Index (CI), is directly proportional to the area covered by migrating/invading cells.

For invasion experiments, 4 h prior to the experiment the transwells inserts of the 16-well CIM Plates were coated with 20 µl of growth-factor-reduced Matrigel (BD Biosciences) at a concentration of 250 µg/ml, whereas for migration experiments the transwells were kept uncoated. The upper chambers were loaded with serum-free medium, and the lower chambers were filled with medium supplemented with 10% FCS (to serve as chemoattractant), both containing either vemurafenib or vehicle. 20,000 cells were seeded in every upper chamber, and allowed to migrate for 12 h. The CI was automatically monitored, with intervals of 30 min within the first 6 h, followed by 60 min intervals afterwards.

### In vitro wound-healing assay

Wound-healing assays were performed by seeding 70,000 cells in each chamber of IBIDI culture-inserts (IBIDI), plated in 24-well dishes. The cells were allowed to grow overnight to form a confluent monolayer. To prevent the influence of cell proliferation, 2 h before the assay the cells were incubated with 10 µg/ml of Mitomycin C. The culture-inserts were then removed, the cells were washed twice with PBS, and serum-supplemented medium (10% FCS) containing vermurafenib or vehicle was applied in each well. Pictures of the wounds were automatically recorded every hour during 48 h using the JuLI Stage real-time cell history recorder system (NanoEnTek).

### Immunoblotting

To prepare total protein extracts for Western blot analysis, the attached cell layer was washed with PBS, and the cell pellets were then collected and lysed by scrapping in 200 µl of 1 × RIPA buffer supplemented with cOmplete EDTA-free protease inhibitor cocktail (Roche). Total cell extracts were quantified with DC Protein Assay (Bio-Rad) and boiled in 1 × Laemmli loading buffer at 95 °C in reducing conditions for 5 min. Samples were frozen at – 80 °C until further use.

For each experimental condition, 20 µg of protein extract were resolved using 12.5% SDS-PAGE, run at 150 V on 1 × TGS buffer (25 mM tris, 192 mM glycine, SDS 0.1%). Proteins were transferred onto methanol-activated PVDF membranes using a Mini-Transblot Cell (Bio-Rad), run at 100 V at 4 °C for 2 h with 1 × transfer buffer (25 mM tris, 200 mM glycine, 20% methanol). Membranes were blocked for 1 h with 5% non-fat milk in 1 × TBST solution (20 mM tris, 150 mM NaCl, 0.1% Tween-20; pH 7.54). Probing with primary antibodies was carried out at 4 °C overnight, with antibody dilutions prepared in 1 × TBST with 0.5% BSA and 0.1% NaN_3_. Antibodies against the following epitopes were used: phospho-MEK1/2 Ser217/221 (1:1000; 41G9; Cell Signaling), MEK1/2 (1:1000; L38C12; Cell Signaling), phospho-ERK1/2 Thr44/Tyr42 (1:1000; 9101; Cell Signaling), and Erk1/2 (1:1000; L34F12; Cell Signaling). Glyceraldehyde 3-phosphate dehydrogenase (GAPDH) (1:3333; 9484; Abcam) served as control for uniform protein loading. After 3 washes with 1 × TBST, the membranes were incubated for 1 h at room temperature with the appropriate HRP-conjugated secondary antibodies, diluted in 1 × TBST: anti-rabbit IgG (1:10,000; NA934V; GE Healthcare) and anti-mouse IgG (1:10,000; NXA931V; GE Healthcare).

Chemiluminescence protein detection was done with SuperSignal West Pico Luminol/Enhancer solution (Thermo Scientific) and the FUSION FX imaging system (Vilber Lourmat). Density of the bands was measured with the Fusion Capt Advance software (Vilber Lourmat) and normalized to GAPDH loading control detected on the same membranes.

### Statistical analysis

Quantification values from at least three independent experiments were used for statistical analysis. Technical replicates within every experiment were averaged and normalization by sum of the replicates [49] was performed to subtract baseline differences between the experiments. The exact number of independent experiments considered for every analysis is given within the figure legends. In experiments comparing the effects of different drug treatments, GraphPad Prism 8 software was used to perform one-way analysis of variance (ANOVA) to evaluate main and interaction effects, followed by Tukey post-test to calculate statistical significance of the magnitude of changes observed between all the different individual experimental conditions. For time-course experiments, two-way ANOVA was used, followed by Sidak post-test to compare the time-matched effects of the drugs. Changes were considered significant at a p-value cutoff of 0.05 in all analyses, unless otherwise stated. All data are presented as the mean ± SEM of the experiments.

## Results

### Dynamic transcriptional changes lead to an enhanced proliferation of fibroblasts after vemurafenib treatment

To determine whether vemurafenib exerts off-target effects that could reshape the tumor microenvironment, we focused on understanding the dynamic transcriptional changes induced in non-transformed human dermal fibroblasts as a major stromal component [15, 16]. A time-course stimulation (4 h, 8 h and 18 h) of juvenile foreskin-derived HDF was carried out with 2 µM of vemurafenib, a standard concentration for in vitro experiments, previously demonstrated to effectively inhibit ERK1/2 signaling in several BRAF^*V600E*^-mutant cells [14, 50]. Transcriptomic changes were assessed in comparison to the corresponding time-matched vehicle-treated (DMSO) controls.

**Fig. 1:**
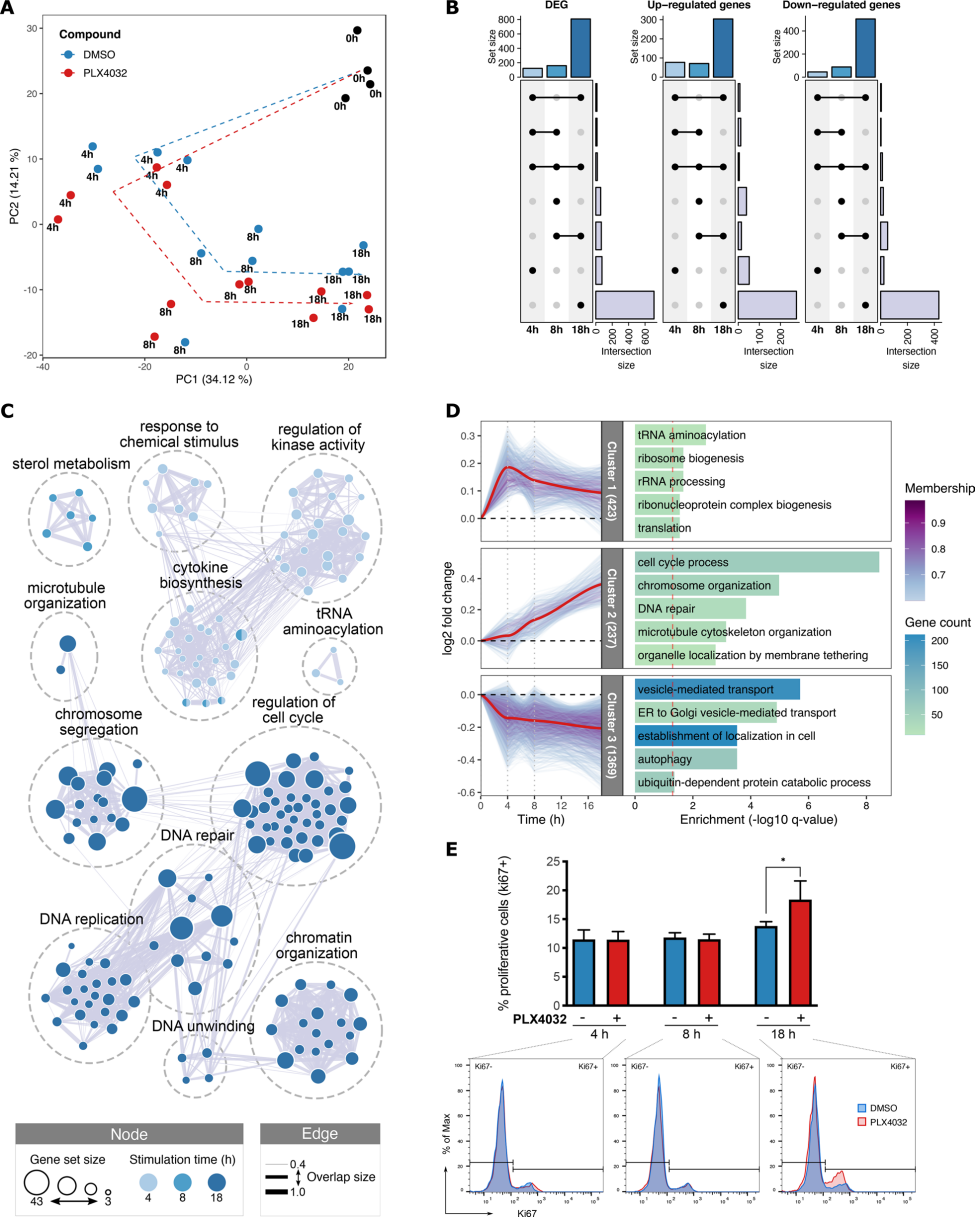
Dynamic transcriptome changes in HDF induced by vemurafenib. Stimulation was done with 2 µM of vemurafenib (PLX4032) or analog volume of vehicle (DMSO) for the indicated duration. **A** Principal component analysis of the transcriptomics datasets in HDF. The eigenvalues from the first two main components (PC1-PC2) are plotted, and the percentage of variance explained by each one is indicated in brackets. Average changes in the expression states are suggested by the dashed trajectories. **B** UpSet plot showing the number of differentially expressed genes (DEG; q < 0.05) in HDF stimulated with vemurafenib in comparison to DMSO control. **C** Significantly over-represented (q < 0.1) biological processes among the genes up-regulated by vemurafenib treatment. Every node represents a single GO term colored by stimulation time, with the node size indicating the number of up-regulated genes within each term. Nodes are connected based on their similarity score (combined Jaccard + Overlap score > 0.4). **D** Soft clustering based on the top 30% of genes with the highest fold changes. The number of genes within every cluster is indicated in brackets; only genes with a minimum membership score of 0.6 are included. Significantly over-represented (q < 0.05) biological processes for every cluster are shown in the adjacent bar plot. **E** Flow cytometry-based determination of the proliferative state. Top: quantification (mean ± SEM) of the percentage of proliferative cells (Ki67 positive) for three independent experiments (n = 3). Bottom: representative histograms showing the frequency distribution of Ki67 levels for the indicated conditions

Principal component analysis (PCA) did not reveal a strong segregation of the transcriptomes based on the drug treatment; rather, the majority of the variance in gene expression was mainly driven by the stimulation time (Fig. 1A), suggesting that the short-term effects of vemurafenib on non-transformed HDF are likely mild. Nevertheless, a small but consistent segregation between vemurafenib- and vehicle-treated HDF was still evidenced over the trajectories of the different stimulation time-points, mainly driven by the second principal component.

Differential gene expression analysis indeed revealed a considerable number of genes (n = 957) in HDF, whose expression was significantly altered over time during vemurafenib treatment. The largest changes were observed after 18 h of stimulation with the inhibitor (Fig. 1B, Additional file 12: Fig. S1A) suggesting a dynamic and progressive effect of the drug.

Over-representation analysis, performed to identify biological processes likely to be affected by vemurafenib, revealed that the early up-regulated genes are mainly involved in the modulation of cytokine biosynthesis and MAPK activity, followed by a latter increase in the expression of genes implicated in several cellular proliferation processes, such as chromatin organization, chromosome segregation, cell cycle, and DNA replication and repair (Fig. 1C, Additional file 12: Fig. S1B, Additional file 8: Table S8). Moreover, vemurafenib treatment also induced a down-regulation of genes involved in biosynthetic process, extracellular matrix (ECM) and cytoskeleton organization, cell adhesion, and others (Additional file 12: Fig. S2, Additional file 9: Table S9).

To further identify dynamic patterns of gene expression changes in HDF induced by vemurafenib treatment, we performed soft clustering of the genes with the highest additive fold changes over time. This method keeps the possibility for every gene to be assigned to multiple clusters at the same time, albeit with different degrees of membership, providing a more noise-robust approach to studying time-course data [51]. The analysis allowed the identification of three main dynamic clusters (Fig. 1D). Two of these comprised genes that were mainly up-regulated after treatment with vemurafenib. Specifically, one of these clusters comprised genes involved in protein biosynthesis processes that show an early up-regulation in their expression profiles. In addition, a gradual increase in the expression of genes involved in cell proliferation was also depicted. A third cluster comprised the largest group of genes (n = 1369), including down-regulated genes mainly involved in autophagy and proteolytic processes, which display an early and sustained reduction of their expression after vemurafenib treatment.

Altogether, the transcriptome analysis suggests the induction of early changes in HDF that begin with an increased expression of genes involved in MAPK signaling and biosynthetic processes, which likely prime and prepare the cells to achieve a subsequent enhanced proliferation. To verify whether vemurafenib treatment induced transcriptional profiles that lead to a higher proliferative state of HDF, intracellular flow cytometry analysis of the proliferation marker Ki67 was performed. We confirmed an increase in the number of cycling cells under vemurafenib stimulation, as revealed by a higher percentage of Ki67 + (non-quiescent) cells. However, in line with the dynamics of the signatures observed at the transcriptomic level, these changes were only detectable after 18 h of stimulation with vemurafenib (Fig. 1E).

Moreover, we addressed whether vemurafenib-induced changes in the expression of genes implicated in cell adhesion and ECM production could be related with alterations in motility behaviors from HDF. RTCA revealed a small but significant increase in HDF migration (chemotaxis), although no evidence was found of an alteration of cell invasion (Additional file 12: Fig. S3A–B). The migratory changes, however, were not recapitulated by wound-healing assays, suggesting that these might be evident only in the presence of a chemotactic gradient that promotes HDF migration (Additional file 12: Fig. S3C).

Given that enhanced proliferative and migratory properties are displayed by activated cancer associated fibroblasts (CAFs) [52], we explored whether the observed vemurafenib-induced changes could reflect an activation of HDF. To this end, gene set enrichment analysis was performed for the tumoral stroma signatures reported by Davidson et al. [53], which comprise signature genes from three distinct fibroblast functional-subpopulations (immune, desmoplastic, and contractile) identified at single-cell resolution from melanoma tumors. A sustained up-regulation of these genes was not observed, with a transient enrichment of contractile and desmoplastic stromal signatures only at 4 h of stimulation with vemurafenib (Additional file 12: Fig. S4). Likewise, we could not detect an enhanced expression of genes encoding for classical CAF markers, such as α-smooth muscle actin (α-SMA), fibroblast activation protein (FAP), fibroblast-specific protein-1 (FSP1), or platelet derived growth factor receptors (PDGFR).

### Vemurafenib prompts transcriptional signatures of paradoxical MAPK/ERK pathway activation in fibroblasts

Based on our results indicating an enhanced proliferative state of HDF after vemurafenib treatment, and given the key role that the MAPK/ERK pathway exerts on the regulation of this cellular process, we investigated whether the observed changes in expression profiles of HDF could be attributed to a direct effect of the drug on the activation state of this pathway. Since paradoxical effects of vemurafenib on BRAF^*WT*^ cells have been reported elsewhere [10, 11, 54, 55], we aimed to contrast the molecular changes observed in BRAF^*WT*^ and BRAF^*V600E*^ cells to identify putative signatures of a paradoxical MAPK/ERK activation.

To this end, we compared the transcriptomics changes in the BRAF^*WT*^ HDF after 18 h of vemurafenib stimulation, with those from two different BRAF^*V600E*^-positive malignant melanoma cell lines (MaMel21 and MaMel63a) [56], treated for 24 h with an equivalent concentration of the inhibitor (2 µM). These melanoma cell lines show different resistance levels to BRAF^*V600E*^ inhibition, with MaMel63a being more prone to induced apoptosis at equimolar concentrations of vemurafenib (Additional file 12: Fig. S5).

**Fig. 2:**
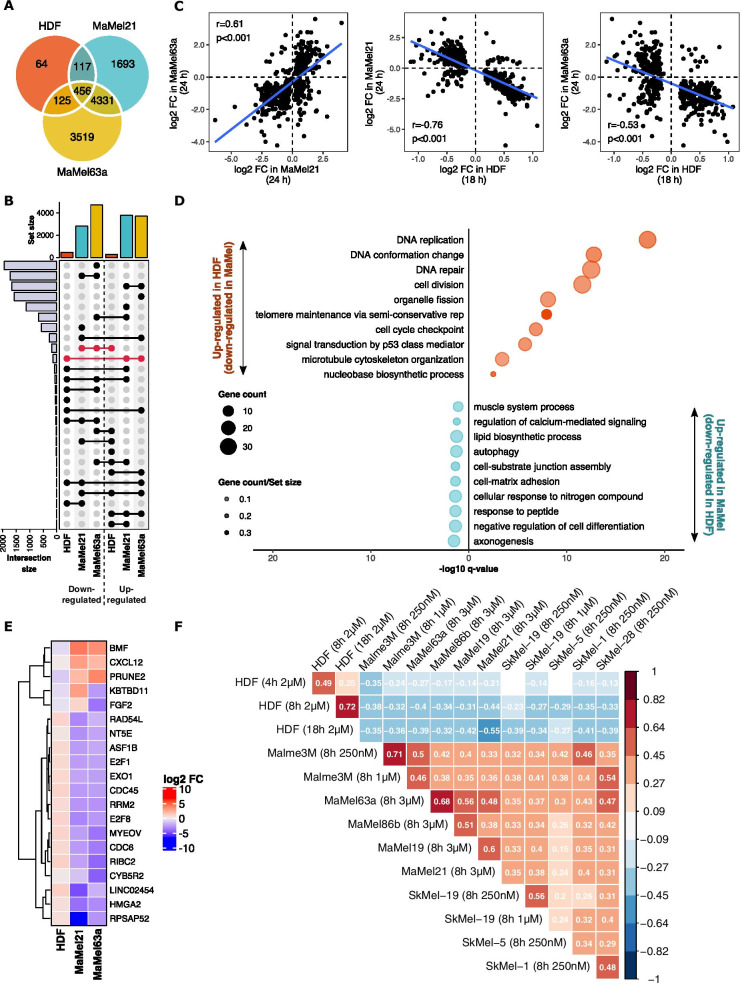
Comparison of the transcriptomic effects of vemurafenib on HDF and MaMel cells. **A** Venn diagram showing the number of DEGs (q < 0.05) on BRAF^*V600E*^ mutant malignant melanoma cells (MaMel21 and MaMel63a) and BRAF^*WT*^ HDF cells after 18 h and 24 h of treatment with 2 µM of vemurafenib, respectively. **B** UpSet plot showing the number of up- and down-regulated genes for each cell line. The subsets of genes that are affected in an inverse direction in HDF and MaMel cells are highlighted in red. **C** Pearson correlation of the log_2_ fold changes (FC) within the subset of commonly deregulated genes in all the studied cell lines (n = 456). The duration of the stimulation for each cell line is indicated in parenthesis. **D** Dot plot of significantly over-represented biological processes (q < 0.1) within the subsets of negatively correlated DEGs in HDF and MaMel cells. The size of every dot represents the number of DEGs within every process. The level of transparency of the dots corresponds to the ratio of the number of DEGs to the total number of genes within every gene set. **E** Heatmap displaying the genes with the highest significant fold changes within the subset of commonly deregulated genes in all the studied cell lines. Fold change values of vemurafenib-treated cells with respect to DMSO control are depicted by a colorimetric scale (red: up-regulated, blue: down-regulated). **F** Correlation matrix of the FC after vemurafenib treatment in different BRAF^*V600E*^ positive melanoma cell lines and HDF, for the subsets of negatively correlated DEGs in HDF and MaMel cells (n = 268). The correlation level is annotated and represented by the colorimetric scale. Only significant correlations (*p* < 0.05) are colored. The stimulation time and inhibitor concentration applied to each cell line is indicated in parenthesis

Differential gene expression analysis of vemurafenib-treated cells relative to DMSO control revealed that, among the genes that were expressed in all the studied cell lines (n = 12,396), a considerably large number were deregulated in MaMel21 (n = 6597) and MaMel63a (n = 8431), whereas fewer genes were significantly affected in HDF (n = 762). Moreover, the majority of the genes whose expression was altered in HDF were also deregulated in the MaMel cells (n = 456; Fig. 2A), although the majority of these changes seemed to occur in opposite directions (Fig. 2B). Pearson correlations of the log_2_ FC for these differentially expressed genes were computed, showing a positive correlation between the changes observed for both melanoma cell lines (r = 0.61, p < 0.001). In contrast, a strong but negative correlation was obtained by comparing the gene expression changes in HDF with those from MaMel21 (r =  − 0.76, *p* < 0.001) and MaMel63a (r =  − 0.53, *p* < 0.001), providing evidence of a major inverse effect of the drug on these cell lines (Fig. 2C).

Within the subset of inversely correlated genes, those that were up-regulated in HDF while down-regulated in melanoma cells (n = 187) upon vemurafenib treatment, mainly comprised genes involved in nucleobase metabolism, DNA organization, replication and repair, cell division, and others, confirming the opposite effect of the drug over the expression of key regulators of cellular proliferation processes, such as E2F transcription factors or cell division control proteins (CDC) among the genes with the highest changes (Fig. 2D–E). Likewise, down-regulated genes in HDF that were up-regulated in melanoma cells (n = 124) were related to biological processes such as cell-substrate junction assembly, cell–matrix adhesion, and regulation of actin assembly, which could impact cell motility. However, these processes seemed to be overall affected in a lower extent by vemurafenib treatment (Fig. 2D).

An inverse regulation of gene expression in HDF upon exposure to vemurafenib was consistently observed when compared to other cell lines harboring the BRAF^*V600E*^ mutation (Fig. 2F). These included two additional MaMel cell lines (MaMel19 and MaMel86b) from our own previously generated dataset, and five melanoma cell lines (Malme3M, SkMel-1, SkMel-5, SkMel-19, and SkMel-28) from the publicly available dataset from Joseph et al. [50], all stimulated for 8 h with different concentrations of vemurafenib, ranging from 250 nM to 3 µM. Regardless of the cell line and the applied inhibitor concentration, similar correlation levels were found for the subset of DEG that were found to be inversely correlated between HDF and MaMel.

To further explore whether the observed effects in HDF could be attributed to a paradoxical activation of the MAPK/ERK pathway, we assessed the expression of genes from the MEK-dependent transcriptional signature reported by Pratilas et al. [57]. This set comprises a total of 52 genes showing significant expression changes after 8 h of MEK inhibition (using PD0325901) in 7 melanoma and 2 colon BRAF^*V600E*^ positive cell lines. From this set of genes, only 4 were reported to be up-regulated after pharmacological inhibition, whereas the remaining genes were 2- to 124-fold down-regulated in the tested cell lines.

**Fig. 3:**
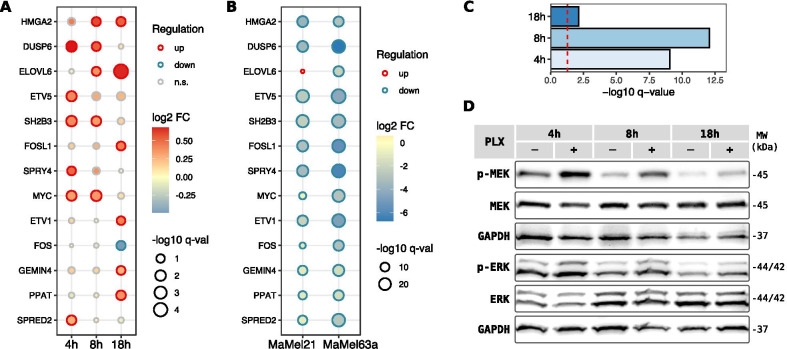
Effects of vemurafenib on the MAPK/ERK pathway activation. **A** Dot plot showing the dynamic expression changes in the MEK-dependent transcriptional signature [57] in HDF after vemurafenib treatment in comparison to DMSO control. Only genes significantly deregulated (q < 0.1) in at least one of the assessed stimulation time-points (4 h, 8 h and 18 h) are plotted, with the direction of the expression changes indicated by the stroke color (red: up-regulated, blue: down-regulated, n.s.: non-significant) and the fold changes (FC) depicted by the colorimetric scale. **B** Dot plot of expression changes in two malignant melanoma cell lines (MaMel21 and MaMel63a) for the MEK-dependent transcriptional signature, after 24 h of vemurafenib treatment. Only genes found to be differentially expressed in HDF after stimulation with the drug are also shown for melanoma cells, with the direction of expression changes indicated by the stroke color (red: up-regulated, blue: down-regulated, n.s.: non-significant) and the FC depicted by the colorimetric scale. **C** Gene set enrichment in HDF for the aforementioned signature for the indicated stimulation time-points. The significance cutoff (q < 0.05) is shown as a dashed red line. **D** Representative Western blot showing the effect of vemurafenib (PLX) treatment over MEK1/2 and ERK1/2 phosphorylation in HDF. The phosphorylated (p-) and total proteins are shown. GAPDH is included as loading control

Out of 50 genes from the MEK-dependent transcriptional signature that were detected as expressed in our datasets, we observed significant expression changes in 13 of these genes after different time-points of stimulation of HDF with vemurafenib. The highest fold changes occurred for the genes encoding for the dual-specificity phosphatase 6 (DUSP6; FC = 0.68), which exerts an inhibitory feedback on the MAPK/ERK signaling, and for the elongation of very long chain fatty acids protein 6 (ELOVL6; FC = 0.65; Fig. 3A), involved in the synthesis of fatty acids. With the only exception of FOS, all the significantly deregulated signature genes were overexpressed in HDF after vemurafenib treatment.

Opposite changes in the expression profiles of these genes were observed for MaMel cells treated with vemurafenib (Fig. 3B), indicating an inverse effect of the drug in the activation state of the signaling pathway. In this case, the observed changes were larger, with the great majority of the signature genes showing a stronger down-regulation (43 and 48 down-regulated genes for MaMel21 and MaMel63a respectively, with changes that reached a seven-fold reduction in expression).

Although the number of significantly deregulated genes from the MEK-dependent transcriptional signature was considerably smaller for HDF, we speculated that modest (below significance threshold), but coordinated changes in expression of these signature genes could be expected. To test this, gene set enrichment analysis was performed, supporting a highly significant up-regulation of the signature (Fig. 3C). Although expression of this signature was enhanced during all the tested time-points, enrichment was stronger for the initial stimulation times, showing a dynamic regulation of the pathway activation.

Western blot analysis confirmed a paradoxical effect of vemurafenib on HDF, evidenced by an increase of MEK1/2 and ERK1/2 phosphorylation in lysates from cells treated with the drug, indicative of an enhanced activation state of the MAPK/ERK signaling pathway (Fig. 3D). Although the basal MEK1/2 and ERK1/2 phosphorylation was strongest at earlier time-points, an hyperphosphorylation of both kinases was detected throughout all the analyzed time-points.

**Fig. 4:**
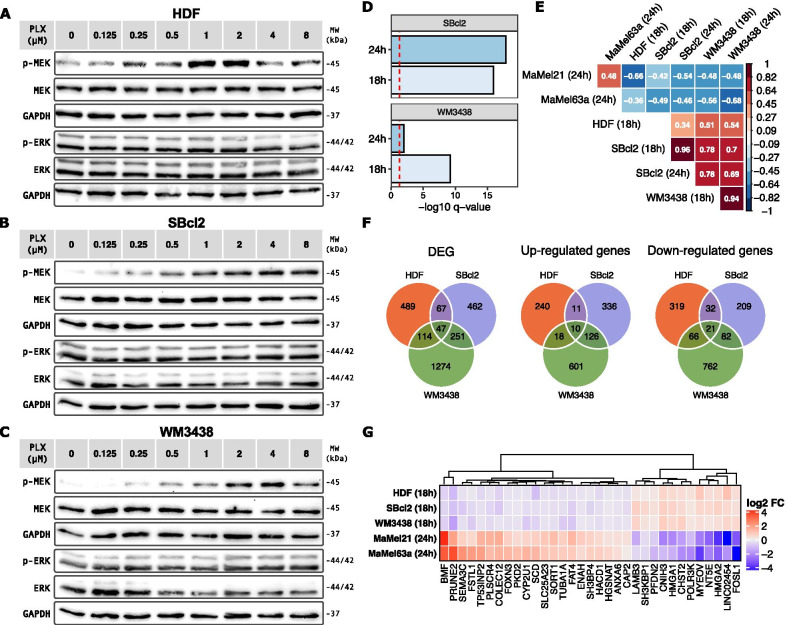
Comparison of the transcriptomic effects of vemurafenib on HDF and BRAF^*WT*^ melanoma cells. Western blot showing the effect of vemurafenib (PLX) over MEK1/2 and ERK1/2 phosphorylation in **A** HDF, **B** SBcl2, and **C** WM3438 cell lines, after 4 h of stimulation with increasing concentrations of the inhibitor. The phosphorylated (p-) and total proteins are shown. GAPDH served as a loading control. **D** Gene set enrichment of the MEK-dependent transcriptional signature [57] in BRAF^*WT*^ melanoma cell lines for the indicated stimulation time-points with 2 µM of vemurafenib. The significance cutoff (q < 0.05) is shown as a dashed red line. **E** Correlation matrix of the FC after vemurafenib treatment in BRAF^*WT*^ (HDF, SBcl2, and WM3438) and BRAF^*V600E*^ positive (MaMel21 and MaMel63a) cell lines, for the subsets of DEGs (*p* < 0.1) in common for all conditions (n = 33). The correlation level is annotated and represented by the colorimetric scale. Only significant correlations (*p* < 0.05) are colored. The stimulation time is indicated in parenthesis. **F** Venn diagram showing the number of DEGs (q < 0.05) on BRAF^*WT*^ cell lines after 18 h of treatment with 2 µM of vemurafenib. **G** Heatmap displaying the genes with a paradoxical expression change, consistent in all assayed cell lines. Fold change values of vemurafenib-treated cells with respect to DMSO control are depicted by a colorimetric scale (red: up-regulated, blue: down-regulated)

To identify the set of genes with the most robust paradoxical response across different genetic backgrounds, we further addressed the transcriptional response to vemurafenib in two BRAF^*WT*^ melanoma cell lines (SBcl2 and WM3438). These cell lines showed an early paradoxical BRAF activation within a similar vemurafenib concentration range as HDF (Fig. 4A–C), with a stepwise MEK1/2 hyperphosphorylation upon increasing inhibitor concentrations, which can be detected both below and above the 2 µM concentration employed in further experiments.

Transcriptomic profiling for BRAF^*WT*^ cell lines was carried out after 18 h and 24 h of vemurafenib treatment, to match the time-points previously assessed for HDF and MaMel cells, respectively. PCA for all the different cell lines assayed in this study showed a main segregation of the transcriptomes based on the cell identity. While it is possible to distinguish vemurafenib- from vehicle-treated profiles for cell lines harboring the BRAF^*V600E*^ mutation (i.e., MaMel21 and MaMel63a), this segregation was not evident for those with wild-type BRAF, indicating that the transcriptomic differences among the cell lines greatly outweigh the modest off-target effects from vemurafenib in BRAF^*WT*^ cells (Additional file 12: Fig. S6A). In line with this, and as observed in HDF, differential gene expression analysis revealed that the number of genes whose expression was significantly altered by vemurafenib was considerably smaller for SBcl2 and WM3438 melanoma cell lines than for those harboring the BRAF^*V600E*^ mutation (Additional file 12: Fig. S6B).

Up-regulated genes in BRAF^*WT*^ melanoma cells were involved in processes such as cell adhesion, extracellular matrix organization, and Ras signaling transduction, while processes related to cell proliferation and biosynthesis were decreased (Additional file 12: Fig. S7A). Intracellular flow cytometry analysis of the proliferation marker Ki67 did not reveal an increase in the number of cycling cells under vemurafenib stimulation as observed for HDF (Additional file 12: Fig. S7B), despite a paradoxical MEK1/2 and ERK1/2 hyperphosphorylation across all the assayed time-points (Additional file 12: Fig. S7C).

Similar to HDF, transcriptome analysis revealed a significant up-regulation of the MEK-dependent signature genes in both BRAF^*WT*^ melanoma cell lines (Fig. 4D). Moreover, the comparison of the changes induced by vemurafenib in the expression levels of genes commonly deregulated in all cell lines (n = 33) further supported an opposite main effect of the inhibitor in BRAF^*V600E*^ mutant and BRAF^*WT*^ cells, evidenced by inverse fold change correlations among them (Fig. 4E). Although the expression of only a small set of genes was significantly affected in HDF as well as SBcl2 and WM3438 cell lines (Fig. 4F), some of these corresponded to genes with the strongest opposite response in HDF and MaMel cell lines (Fig. 2E and Fig. 4G). These included genes such as the BCL2 modifying factor (BMF), prune homolog 2 with BCH domain (PRUNE2), 5'-nucleotidase ecto (NT5E, or CD73), myeloma overexpressed (MYEOV), high mobility group A2 (HMGA2), and the long intergenic non-protein coding RNA 2454 (LINC02454), which consistently showed a paradoxical response in all the BRAF^*WT*^ cells.

### Permissive chromatin landscapes allow early vemurafenib-induced transcriptome changes

Given that previous studies have provided evidence that inhibition of the MAPK/ERK pathway can trigger a remodeling of the chromatin regulatory architecture [58], we investigated whether this could underly some of the observed vemurafenib-induce transcriptional changes in HDF, which could hint to the occurrence of epigenetically-mediated longer-lasting side effects of the drug on the stromal cells. To this end, a time-course stimulation of HDF with vemurafenib or DMSO was performed in the same way as in the RNA-seq experiments, and the chromatin accessibility landscapes were profiled through ATAC-seq.

**Fig. 5:**
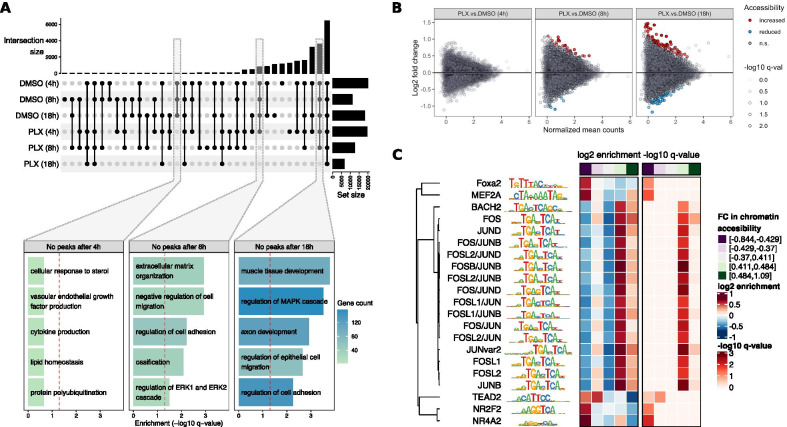
Chromatin accessibility profiles in HDF after vemurafenib treatment. Stimulation was done with 2 µM of vemurafenib (PLX4032, or PLX for short), or analog volume of vehicle (DMSO), for the indicated duration. **A** The UpSet plot shows the number of accessibility peaks identified for each condition. The bar plot below shows the annotation of over-represented biological processes for the genes within the subsets of peaks that are not detected after different time-points of stimulation with vemurafenib. The significance threshold for biological over-representation (q < 0.05) is indicated by the dashed red line. **B** Scatter plot showing the fold changes (FC) in chromatin accessibility for 21,800 peaks after vemurafenib treatment in comparison to the corresponding time-matched DMSO control. The FDR adjusted significance of the changes (q-value) for every region are represented by the level of transparency of the corresponding points, with significantly increased or reduced accessibility regions (q < 0.1) colored in red and blue, respectively. **C** Enriched transcription factor binding motifs within promoter peaks, after 18 h of stimulation with vemurafenib in comparison to DMSO control. Enrichment is estimated for binned sets of peaks, based on their FC in chromatin accessibility, as indicated by the top annotation of the heatmaps (purple: bins of peaks with higher accessibility under DMSO, green: bins of peaks with higher accessibility under vemurafenib). The heatmaps display the levels of enrichment (left) and significance (right) for each motif. The dendrogram represents the clustering of the motifs based on their sequence similarity

The comparison of the coverage of ATAC-seq reads within promoter regions showed an overall concordance with the levels of gene expression, with the majority of the non-expressed genes in HDF showing low promoter accessibility (Additional file 12: Fig. S8). Nevertheless, we were not able to identify any set of promoter regions with a clear different accessibility pattern across the experimental treatments. Moreover, the comparison of accessibility peaks for vemurafenib- and vehicle-treated HDF showed that the great majority of peaks are shared among the conditions (intersection size = 6414; Fig. 5A); nevertheless, a reduction in the overall number of accessibility peaks was observed along vemurafenib treatment. Functional annotation revealed that peaks identified for DMSO control, which were not detected after 8 h or 18 h of vemurafenib treatment, were located within or nearby genes involved in cell migration and adhesion, as well as MAPK signaling cascade, mirroring some of the mainly altered processes at the transcriptome level (Fig. 5A, lower panel).

To obtain a more quantitative determination of the regions that undergo changes in chromatin accessibility after vemurafenib treatment, we performed differential accessibility analysis within the identified ATAC-seq peak regions. We could observe a small, but increasing number of DARs over time (Fig. 5B; Additional file 10: Table S10). Although we could consistently find that some of these DARs are located within or in the proximity of genes involved in cell adhesion and MAPK/ERK signaling cascade, we could not detect a significant enrichment of peaks located in genes involved in these processes (Additional file 11: Table S11). By looking for enrichment of TF binding motifs within the regions with highest accessibility changes, we could determine that the promoter peaks that seemed more accessible (log_2_ FC > 0.4) after 18 h of vemurafenib treatment were enriched in AP-1 (FOS/JUN) binding motifs (Fig. 5C). However, it has to be noted that the changes in these regions are only below the significance threshold (q > 0.1).

**Fig. 6:**
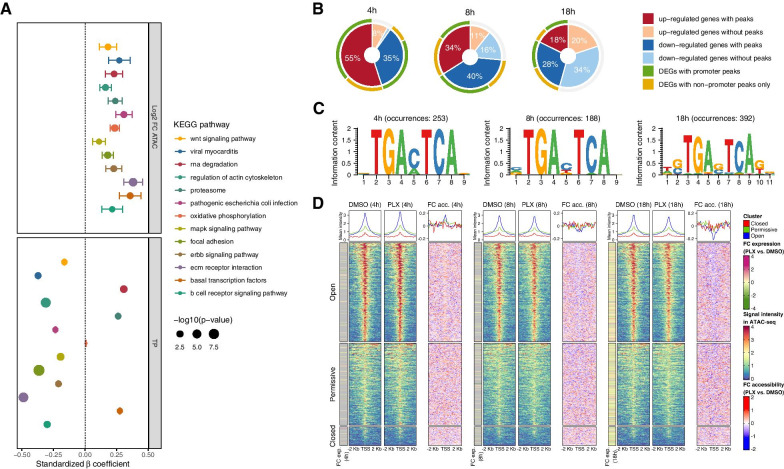
Relation of chromatin accessibility and gene expression changes in HDF after vemurafenib treatment. **A** Multivariate regression models for gene-sets (KEGG pathways) showing a significant relation between gene expression changes after vemurafenib treatment, with the changes in accessibility of promoter peaks (log_2_ FC ATAC), and the length of stimulation (TP). The plot shows the standardized β coefficients of the independent variables for each regression models. The size of every dot represents the significance of each coefficient, and the whiskers indicate the SE. The plot only shows those gene-sets where the model, and the β coefficients are significant (q < 0.05, and *p* < 0.05, respectively). **B** Distribution of accessibility peaks within DEGs (q < 0.05), after different durations (indicated on top of the plot) of stimulation with vemurafenib in comparison to DMSO control. **C** Sequence logo for the motifs identified within accessibility peaks located in, or nearby DEGs. The stimulation time with vemurafenib, and the number of occurrences of each motif is indicated on top of the logo. **D** Accessibility within promoter regions and transcription start sites (TSS) of DEGs (q < 0.05). For each stimulation time-point (indicated in brackets) the heatmaps display the coverage of ATAC-seq reads (left, and middle), and the fold changes in accessibility (FC acc.) in vemurafenib- (PLX, for short) treated HDF, compared to DMSO control. The FC in gene expression (FC exp.) is indicated as a left annotation for each stimulation time-point. Top annotations show the mean coverage along promoter regions for each of the defined clusters (red: closed, green: permissive, blue: open)

We sought to determine whether the observed changes in chromatin accessibility could be underlying some of the transcriptional alterations detected during vemurafenib treatment. An overall poor correlation was observed, with few overlapping DARs and DEGs (Additional file 12: Fig. S9). Moreover, ROC and PR curves showed a low predictive ability of the changes in promoter accessibility over the alterations in gene expression (Additional file 12: Fig. S10), suggesting that the overall gene expression changes in HDF that occur after vemurafenib treatment are not greatly determined by the modulation of promoter accessibility. However, for specific pathways, such as those involved in cell adhesion, interactions with ECM, and regulation of actin cytoskeleton, we could establish a small but direct relation between gene expression and promoter accessibility changes after treatment with vemurafenib, as depicted by the positive β coefficients in multivariate regression models (Fig. 6A, Additional file 12: Fig. S11).

As transcriptional changes in HDF did not seem to be strongly determined by alterations in chromatin accessibility, we investigated whether an already permissive chromatin landscape could facilitate the observed gene expression changes, by allowing the accessibility to MAPK/ERK-regulated transcription factor binding sites. By looking at the distribution of accessibility peaks within DEGs, we could observe that the majority of genes whose expression was altered after 4 h of vemurafenib treatment showed accessibility peaks in promoter or distal regions (Fig. 6B), where AP-1 transcription factor binding motifs were found as particularly abundant (Fig. 6C). For later stimulation time-points, however, the proportion of DEGs with identified peaks was smaller, suggesting that early response genes might rely on a more accessible chromatin state than the late response ones. Despite the lack of identified peaks suggesting a strong accessibility for some of the late response DEGs, the observation of the coverage of ATAC-seq reads within promoter regions showed a distribution of reads compatible with an open or permissive TSS for most of the HDF genes whose expression was altered by vemurafenib (Fig. 6D).

### Downstream BRAF inhibition fails to block the paradoxical effects of vemurafenib

To explore whether combination therapies could offer an advantage in restraining the effects of a paradoxical MAPK/ERK pathway activation in HDF, we determined whether the observed changes could be limited by inhibition of downstream effectors of BRAF. To this end, we treated the cells with vemurafenib in combination with 5 nM of trametinib, an FDA-approved MEK inhibitor, and compared it to each inhibitor applied individually. Previous studies have shown for tumor cells that co-treatment with this drug concentration effectively abolishes the upregulation of ERK1/2 phosphorylation induced by BRAF inhibitors alone (King et al., 2013).

**Fig. 7:**
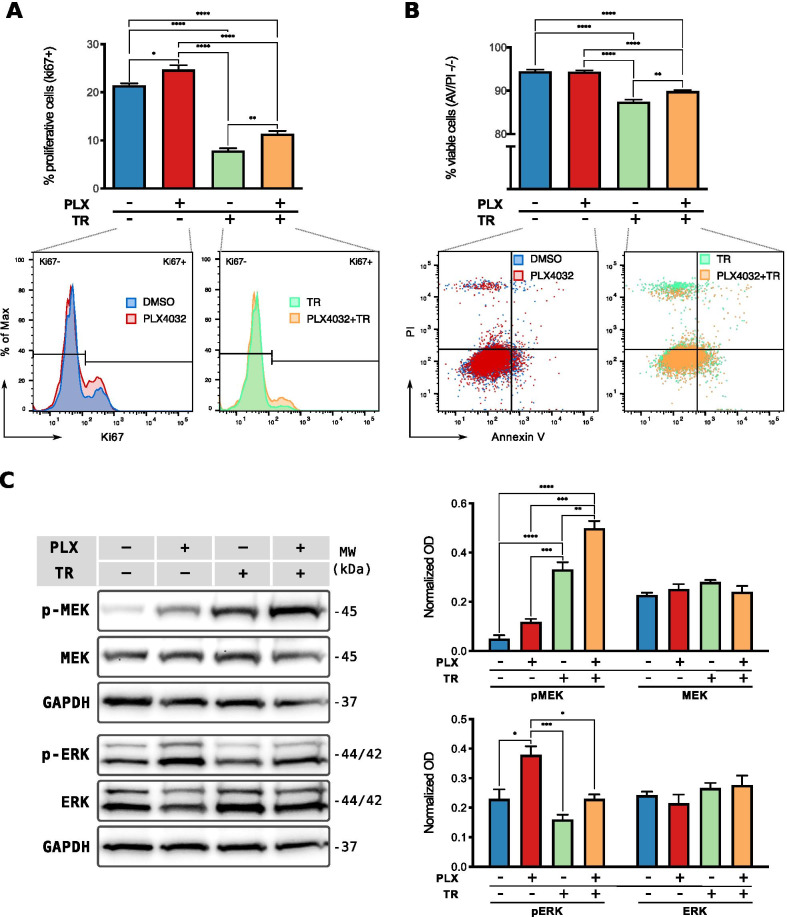
Combined inhibition of MAPK/ERK pathway in HDF. HDF were stimulated with 2 µM of vemurafenib (PLX4032, or PLX for short), 5 nM of trametinib (TR), or the combination. **A** Flow cytometric analysis of the proliferative state after 18 h of inhibition. Top: quantification of the percentage of proliferative cells (Ki67 positive) for four independent experiments (n = 4). Bottom: representative histograms showing the frequency distribution of Ki67 levels for the indicated condition. **B** Flow cytometric analysis of cellular viability after 66 h of inhibition. Top: quantification of the percentage of viable cells (Annexin V–PI negative) for four independent experiments (n = 4). Bottom: Representative scatter plots showing the viable (lower left quadrant) and apoptotic/necrotic cells. **C** MEK1/2 and ERK1/2 phosphorylation after 18 h of inhibition. Right: representative Western blot for the phosphorylated (p-) and total proteins. GAPDH is shown as loading control. Left: densitometry quantification for three independent experiments (n = 3). Optical density (OD) was normalized to the corresponding loading control. All bar plots show the mean ± SEM of the experiments. *p < 0.05, **p < 0.01, ***p < 0.001, ****p < 0.0001

We first investigated the impact on the proliferative state of HDF, 18 h after stimulation with the inhibitors, and found that trametinib on its own leads to a significant reduction in the percentage of cycling cells. However, the observed effect was slightly attenuated when administered in combination with vemurafenib. Accordingly, a higher percentage of proliferative cells was detected in cultures stimulated with the drug combination than in those treated with trametinib alone, indicating that a paradoxical induction of proliferation by vemurafenib still occurred under MEK inhibition (Fig. 7A).

We further explored the impact of the drug combination in HDF viability using flow cytometry-based detection of cellular apoptosis/necrosis. After 66 h of stimulation, we observed no effect on cell viability under vemurafenib alone, whereas it was slightly impaired by MEK inhibition with trametinib. However, in line with proliferation experiments, combined stimulation with vemurafenib led to an improved survival of cells under MEK inhibition (Fig. 7B).

To address the activation state of the MAPK/ERK pathway on the protein level, we performed Western blot analysis of MEK1/2 and ERK1/2 phosphorylation. The analysis revealed that 18 h-inhibition with trametinib lead to a strong hyperphosphorylation of MEK1/2 in HDF and, therefore, incomplete inhibition of ERK1/2 (Fig. 7C). Although the same trametinib concentration induced a reduction of MEK1/2 phosphorylation in the melanoma cells and effectively inhibited ERK1/2 (Additional file 12: Fig. S12A), a reduction of ERK1/2 phosphorylation in HDF was mainly effective at earlier time-points (4 h), but partially compensated afterwards through MEK1/2 hyperphosphorylation (Additional file 12: Fig. S12B). Moreover, when combined with vemurafenib, trametinib further enhanced the paradoxical hyperphosphorylation on MEK1/2 observed at 18 h, and hence, no downstream changes in ERK1/2 phosphorylation could be clearly detected (Fig. 7C).

### Paradoxical effects on fibroblasts are restrained by the new generation of BRAF inhibitors

In a next step, we assessed whether the observed paradoxical effects of vemurafenib on HDF could be restrained by the use of a new generation of BRAF inhibitors [59]. These inhibitors (PLX7904, and PLX8394), dubbed “paradox breakers”, have been shown to inhibit MAPK/ERK signaling in melanoma cells with BRAF mutations, without further inducing the pathway in cells bearing upstream activation. We focused on addressing the effects of PLX8394, which is currently being evaluated as a single agent in phase I/IIa clinical trials for patients with BRAF-mutated advanced unrespectable solid tumors (ClinicalTrials.gov, NCT02428712) [60]. This inhibitor was used at a concentration analogous to the one used in experiments with vemurafenib (2 µM), to allow the comparison of the effects of both BRAF inhibitors at equimolar concentrations.

**Fig. 8:**
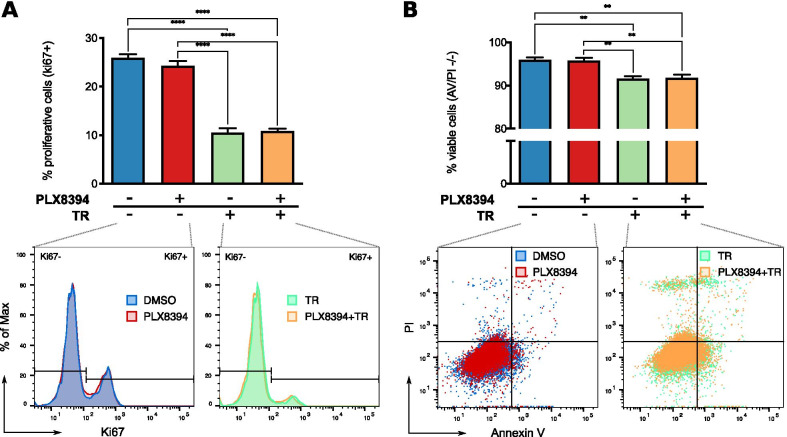
Effect of PLX8394 “paradox breaker” on HDF. HDF were stimulated with 2 µM of PLX8394, 5 nM of trametinib (TR), or the combination. **A** Flow cytometric analysis of the proliferative state after 18 h of inhibition. Top: quantification of the percentage of proliferative cells (Ki67 positive) for four independent experiments (n = 4). Bottom: representative histograms showing the frequency distribution of Ki67 levels in the indicated condition. **B** Flow cytometric analysis of the cell viability after 66 h of inhibition. Top: quantification of the percentage of viable cells (Annexin V–PI negative) for four independent experiments (n = 4). Bottom: Representative scatter plots showing the viable (lower left quadrant) and apoptotic/necrotic cells. All bar plots show the mean ± SEM of the experiments. **p < 0.01, ****p < 0.0001

Treatment of HDF with PLX8394 as a single agent, did not lead to an increase in the proliferative state of the cells, as it was observed for vemurafenib (Fig. 8A). In addition, the viability of the cells stimulated with the new generation of BRAF inhibitor was not affected (Fig. 8B). Moreover, combined stimulation of HDF with PLX8394 and trametinib further evidenced the absence of paradoxical effects, with no detectable counteraction of the impairments induced by trametinib over cell proliferation and viability (Fig. 8A-B), suggesting that the paradox breakers might be potentially advantageous for stroma cells mainly when implemented as monotherapy in the absence of additional MAPK/ERK pathway inhibitors.

## Discussion

The tumor microenvironment is considered a key player during the oncogenic development and progression [61]. Although targeted therapies are selectively aimed at tumor cells, off-side effects are expected. This represents a latent risk of inducing changes in the non-transformed stromal cells, which could further foster cancer progression. However, the direct molecular impact of therapies over the tumor microenvironment is often not addressed in great detail. Fibroblasts in particular frequently make up a major part of the stroma, and their involvement in cancer initiation and progression has been well documented for several tumor types with high prevalence of BRAF mutations [15]. Herein, we profiled the dynamic transcriptional and chromatin accessibility changes that occur in non-transformed HDF upon stimulation with the BRAF^*V600E*^ inhibitor vemurafenib, and explore the potential use of alternative therapies to overcome some of the off-target effects.

We report that vemurafenib induces a sequential activation of transcriptional signatures in HDF which are converse to those of BRAF^*V600E*^ mutant melanoma cell lines, consistent with a paradoxical hyperactivation of the MAPK/ERK pathway in HDF. Our findings are in agreement with previous descriptions of a paradoxical modulation of RAF inhibitors over the activation state of the MAPK/ERK cascade in BRAF^*WT*^ cells [6, 11, 54, 62, 63]. Mechanistically, this has been explained by binding of the inhibitors to one of the protomers of wild-type RAF dimers, inducing a conformational change in the partner drug-free protomer, and its consequent allosteric activation [11, 63, 64].

The molecular outcome of this paradoxical transactivation has been observed in more detail for keratinocytes and BRAF^*WT*^ melanoma cells, as it is thought to be related to the appearance of treatment-emergent secondary skin lesions, mainly comprising melanomas, keratoacanthomas, and squamous cell carcinomas [65, 66]. In line with our findings for HDF, an induction of MEK and ERK phosphorylation as well as an increased proliferative state were also described in these cell types [10, 11, 50, 54]. Investigations of the effects of BRAF inhibitors on fibroblasts have been more sparse and contradictory, with some studies supporting a paradoxical activation [55], while others reporting no enhanced activity in normal fibroblasts [67]. However, these studies have focused on addressing the impact of the drug in specific features of the fibroblasts, such as the release of specific growth factors or ECM production, which might partially account for the contradictory reports.

Through a more unbiased approach, our study provides evidence of a dynamic paradoxical activation of the MAPK/ERK pathway in HDF. We report a significant increase in the expression of MEK-dependent genes, whose induction was strongest at the earliest time-points of simulation with vemurafenib (4 h and 8 h). Likewise, previous studies in BRAF^*WT*^ tumor cells have reported that the paradoxical expression of these genes returns to a baseline level after 8 h of vemurafenib treatment, despite the levels of p-MEK and p-ERK remaining elevated [50]. This transient up-regulation of MEK-dependent signature genes probably reflects a rapid activation of negative feedbacks, such as those exerted by DUSP6 and the sprouty RTK signaling antagonist 4 (SPRY4) which we found to be strongly modulated by the early vemurafenib treatment, in line with previous reports [50, 57].

In spite of this, the greatest vemurafenib-induced transcriptional changes in HDF could be detected at the latest stimulation time-point (18 h), suggesting that the direct and/or indirect transcriptional modulation of MAPK/ERK pathway remains in effect. Not unexpectedly, the transcriptomic changes we detected in HDF were modest in comparison to those in BRAF^*V600E*^ melanoma cell lines, provided the lower binding affinity of the inhibitor to BRAF^*WT*^ [6]. Nevertheless, the off-target effects in HDF seemed pleiotropic, impacting the expression of genes involved in multiple cellular functions.

Particularly prominent was the induction of a higher proliferative state in HDF, as expected from the main role of the MAPK/ERK pathway in the modulation of cell proliferation. This was also accompanied by a sustained reduction in the expression of genes implicated in autophagy, consistent with previous reports showing that MEK/ERK inhibition leads to an increased autophagic flux through the activation of the LKB1/AMPK/ULK1 signaling axis [68]. Moreover, signatures of reduced cell adhesion were also evidenced upon the paradoxical induction of the MAPK/ERK signaling, which might help to explain why vemurafenib has been formerly shown to enhance migration of keratinocytes [54].

The paradoxical effects of BRAF inhibitors have been previously related to the occurrence of treatment-emergent lesions in patients [65, 66]. Moreover, it has been also shown that BRAF^*V600E*^ melanoma cell lines rapidly become tolerant to PLX4720 in areas of high stromal density, prompted by an increase of matrix remodeling induced by treatment with the inhibitor [67]. Whether the vemurafenib-induced changes we report here could actually have an impact on cancer development and progression would still need to be verified. The sole increase in HDF proliferation, leading to a higher fibroblast density within the tumor microenvironment might not necessarily be regarded as a tumor-promoting feature itself, given that the precise role of fibroblast in cancer progression is still debated, with contrasting reports supporting both pro-tumorigenic and anti-tumorigenic roles [69].

However, our data reveal additional vemurafenib-induced changes in HDF which could be further speculated to promote cancer progression and therapeutic escape. Along this line, the antagonistic regulation of genes involved in the modulation of paracrine communication imply that the inhibition of the signaling from tumor cells could be partially compensated by the off-target effects of vemurafenib in the stroma. For instance, an unfavorable impact can be deduced from the up-regulation in HDF of NT5E, provided that it generates immunosuppressive adenosine, and has been linked to immune evasion in melanoma [70]. Moreover, remodeling of cell adhesion could facilitate a collective migration of tumor and stroma cells. Although it has been shown that fibroblast survival is widely dependent on attachment to ECM [55], a reduced adhesion could be supported by the paradoxical down-regulation of factors implicated in anoikis, such as BMF, which is normally released in response to loss of adhesion preceding anoikis and prevents detached cells from colonizing elsewhere [71]. These observations will warrant further research.

Although we found that a main consequence of the off-target effects from vemurafenib is the induction of a less quiescent state in HDF, and despite fibroblast transformation into CAFs being frequently preceded by the acceleration of their proliferation [52], our results argue against a persistent epigenetically-mediated activation of fibroblasts. Typical CAF markers were not altered in their expression levels upon vemurafenib treatment, nor could we identify a clear sustained up-regulation of signatures of tumoral stroma. Although the lack of enrichment of these signatures might account for the recognized heterogeneity of CAFs, which challenges the identification of universal fibroblast-activation markers [72], this most likely reflects that vemurafenib only induces transient transcriptional changes in HDF.

This notion is further supported by the fact that only a few of the expression changes were consistent with a clear alteration of chromatin accessibility, suggesting that an epigenetic modulation might not be a key regulatory layer underlying the main transcriptional changes in our model. Conversely, an already permissive chromatin landscape in fibroblasts seems to allow the early access of MAPK/ERK-regulated transcription factors to key regulatory regions, such as AP-1 binding sites. Although this suggests that vemurafenib-induced long-lasting effects, persistent in the absence of the drug, might be unlikely, our study was limited to exploring the early effects of acute stimulation with vemurafenib. Of note, a significant paradoxical increase in the expression of genes encoding for architectural proteins, such as HMGA2 and anti-silencing function 1B histone chaperone (ASF1B), was detected at the latest time-point of vemurafenib stimulation. Therefore, the impact of a sustained exposure to the inhibitor would still need to be addressed, given the role of these factors in modulation of cell proliferation, DNA repair, apoptosis, and other relevant processes [73, 74].

Altogether, our results raise considerations for the therapeutic use of vemurafenib as single-agent to target the mutated BRAF. We asses for the first time the effects of the new RAF inhibitor PLX8394 on normal fibroblasts, and provide evidence supporting its use to avoid the paradoxical induction of HDF proliferation. The use of this inhibitor as a monotherapy might offer the advantage of avoiding a paradoxical activation of the MAPK/ERK pathway within the tumor stroma, provided its ability to disrupt BRAF-containing dimers [75]. However, we envision that combination therapies with MEK or ERK inhibitors might still provide the advantage to better overcome acquired resistance to RAF inhibitors, arising from the reactivation of the MAPK/ERK pathway by mechanisms such as the disruption of negative feedback elements [76], alterations that promote RAF dimerization [77], downstream activating mutations in MEK [78, 79], and others. In the context of dual inhibition, we believe that the paradoxical effects of vemurafenib could actually help to attenuate the toxicity of less selective MEK/ERK inhibitors in the stromal cells, as we have shown here for the MEK inhibitor trametinib, whose impact on HDF viability and proliferation was partially antagonized by vemurafenib. Future in vivo experiments and clinical trials would be required to evaluate this and identify the therapy that best restrains the tumor growth without compromising normal cells.

## Conclusions

Our study highlights the relevance of assessing the off-target effects induced by specific drug combinations in the stroma cells during cancer treatment. We show that vemurafenib prompts a paradoxical hyperactivation of the MAPK/ERK pathway in HDF. Together with a permissive chromatin landscape, this elicits an early up-regulation of MEK-dependent genes, subsequently impacting gene expression programs involved in multiple cellular functions, including proliferation, autophagy, and cell adhesion. While this raises considerations for the therapeutic use of vemurafenib as a single-agent, and despite as showing that newer BRAF inhibitors can evade some of the off-target effects, the paradoxical outcomes of vemurafenib might help to attenuate the toxicity in the stromal cells when used in combination with less selective MEK/ERK inhibitors. Raising efforts to further understand the impact of targeted therapies on the microenvironment could contribute to better treatment decisions.

## Supplementary Information

**Additional file 1: Table S1**. Gene expression (log_2_ CPM) in HDF treated with 2 µM of vemurafenib or DMSO during 4 h, 8 h, or 18 h.

**Additional file 2: Table S2**. Gene expression (log_2_ CPM) in MaMel21 and MaMel63a treated with 2 µM of vemurafenib or DMSO during 24 h.

**Additional file 3: Table S3**. Gene expression (log_2_ CPM) in SBcl2 and WM3438 treated with 2 µM of vemurafenib or DMSO during 18 h or 24 h.

**Additional file 4: Tables S4A–C**. Differentially expressed genes in HDF treated with 2 µM of vemurafenib, in comparison to the corresponding time-matched DMSO control.

**Additional file 5: Tables S5A–B**. Differentially expressed genes in MaMel21 and MaMel63a treated with 2 µM of vemurafenib, in comparison to DMSO control.

**Additional file 6: Tables S6A–D**. Differentially expressed genes in SBcl2 and WM3438 treated with 2 µM of vemurafenib, in comparison to the corresponding time-matched DMSO control.

**Additional file 7: Tables S7A–F**. ATAC-seq peaks in HDF treated with treated with 2 µM of vemurafenib or DMSO during 4 h, 8 h or 18 h.

**Additional file 8: Tables S8A–C**. Over-represented biological processes among the up-regulated genes in HDF after vemurafenib treatment, in comparison to the corresponding time-matched DMSO control.

**Additional file 9: Tables S9A–C**. Over-represented biological processes among the down-regulated genes in HDF after vemurafenib treatment, in comparison to the corresponding time-matched DMSO control.

**Additional file 10: Tables S10A–B**. Differentially accessible regions in HDF treated with 2 µM of vemurafenib, in comparison to the corresponding time-matched DMSO control.

**Additional file 11: Tables S11A–B**. Over-represented biological processes among genes with differentially accessible regions in HDF after vemurafenib treatment, in comparison to the corresponding time-matched DMSO control.

**Additional file 12: Figs. S1–12**. Figure S1: Differentially expressed genes in HDF after vemurafenib treatment. Figure S2: Down-regulated processes in HDF by vemurafenib treatment. Figure S3: Effect of vemurafenib on HDF motility. Figure S4: Enrichment of tumoral stroma signatures in HDF after vemurafenib treatment. Figure S5: Effect of vemurafenib on MaMel viability. Figure S6: Comparison of transcriptional profiles in BRAF^*WT*^ and BRAF^*V600E*^ mutant cell lines after vemurafenib treatment. Figure S7: Effect of vemurafenib on BRAF^*WT*^ melanoma cell lines. Figure S8: Accessibility within promoter regions and transcription start sites (TSS) in HDF. Figure S9: Pearson correlation of chromatin accessibility and gene expression changes in HDF after vemurafenib treatment. Figure S10: Receiver operating characteristic (ROC) and precision-recall (PR) curves. Figure S11: Most significant gene-sets (KEGG pathways) showing a relation between promoter accessibility and gene expression changes after vemurafenib treatment in HDF. Figure S12: Effect of trametinib on MAPK/ERK pathway activation.

## Data Availability

RNA- and ATAC-seq datasets from this study are available through the NCBI Gene Expression Omnibus (GEO) database, with the accession number GSE188470.

## Abbreviations

ANOVA: Analysis of variance
ASF1B: Anti-silencing function 1B histone chaperone
ATAC: Assay for transposase-accessible chromatin
BMF: BCL2 modifying factor
BRAF: B-rapidly accelerated fibrosarcoma
CAF: Cancer associated fibroblast
CDC: Cell division control protein
CI: Cell index
CPM: Count-per-million
DAR: Differentially accessible region
DEG: Differentially expressed gene
DKFZ: German Cancer Research Center
DMSO: Dimethyl sulfoxide
DUSP6: Dual-specificity phosphatase 6
ECM: Extracellular matrix
ELOVL6: Elongation of very long chain fatty acids protein
ERK: Extracellular signal-regulated kinase
FAP: Fibroblast activation protein
FC: Fold changes
FDR: False discovery rate
FSP1: Fibroblast-specific protein-1
GAPDH: Glyceraldehyde 3-phosphate dehydrogenase
GO-bp: Gene ontology biological processes
HDF: Human dermal fibroblast
HMGA2: High mobility group A2
IC50: Half maximal inhibitory concentration
LINC02454: Long intergenic non-protein coding RNA 2454
MaMel: Malignant melanoma
MAPK: Mitogen-activated protein kinase
MCL: Markov cluster algorithm
MEK: Mitogen-activated protein kinase kinase
MYEOV: Myeloma overexpressed
NT5E, or CD73: 5'-Nucleotidase ecto
PCA: Principal component analysis
PDGFR: Platelet derived growth factor receptors
PFS: Progression-free survival
PI: Propidium iodide
PR: Precision-recall curve
PRUNE2: Prune homolog 2 with BCH domain
ROC: Receiver operating characteristic curve
RTCA: Real-time cell analysis
SEM: Standard error of mean
SPRY4: Sprouty RTK signaling antagonist 4
TF: Transcription factor
TMM: Trimmed Mean of M-values
TSS: Transcriptional start site
V600E: Valine by glutamic acid substitution at codon 600
α-SMA: α-Smooth muscle actin
