# Supplementary material for: Dynamic transcriptome analysis reveals signatures of paradoxical effect of vemurafenib on human dermal fibroblasts
Source: Cell Commun Signal. 2021 Dec 20;19:123. doi: 10.1186/s12964-021-00801-3 (PMC8686565; doi:10.1186/s12964-021-00801-3)
Supplement: Supplementary file 13 — Additional file 12: Figs. S1–12. Figure S1: Differentially expressed genes in HDF after vemurafenib treatment. Figure S2: Down-regulated processes in HDF by vemurafenib treatment. Figure S3: Effect of vemurafenib on HDF motility. Figure S4: Enrichment of tumoral stroma signatures in HDF after vemurafenib treatment. Figure S5: Effect of vemurafenib on MaMel viability. Figure S6: Comparison of transcriptional profiles in BRAFWT and BRAFV600E mutant cell lines after vemurafenib treatment. Figure S7: Effect of vemurafenib on BRAFWT melanoma cell lines. Figure S8: Accessibility within promoter regions and transcription start sites (TSS) in HDF. Figure S9: Pearson correlation of chromatin accessibility and gene expression changes in HDF after vemurafenib treatment. Figure S10: Receiver operating characteristic (ROC) and precision-recall (PR) curves. Figure S11: Most significant gene-sets (KEGG pathways) showing a relation between promoter accessibility and gene expression changes after vemurafenib treatment in HDF. Figure S12: Effect of trametinib on MAPK/ERK pathway activation. [file 12964_2021_801_MOESM13_ESM.pdf]

Supplementary figures for

Dynamic transcriptome analysis reveals signatures of paradoxical effect of vemurafenib on human dermal fibroblasts

Eyleen Corrales[1,2,3], Ella Levit-Zerdoun[1,2,4], Patrick Metzger[2], Silke Kowar[1,2], Manching Ku[6], TilmanBrummer[1,4,5,7] and Melanie Boerries[1,2,4,5,7*]

[1] Institute of Molecular Medicine and Cell Research (IMMZ), University of Freiburg, Stefan-Meier-Str. 17, 79104 Freiburg, Germany.

[2] Institute of Medical Bioinformatics and Systems Medicine (IBSM), Medical Center-University of Freiburg, Faculty of Medicine, University of Freiburg, Breisacherstr. 153, 79110 Freiburg, Germany.

[3] Faculty of Biology, University of Freiburg, Schänzlestr. 1, 79104 Freiburg,Germany.

[4] German Cancer Research Center (DKFZ), Im Neuenheimer Feld 280, 69120 Heidelberg, Germany.

[5] German Cancer Consortium (DKTK), Freiburg, Germany.

[6] Department of Pediatrics and Adolescent Medicine, Division of Pediatric Hematology and Oncology, Medical Center-University of Freiburg, Faculty of Medicine, University of Freiburg, Mathildenstr. 1, 79106 Freiburg, Germany.

[7] Centre for Biological Signalling Studies (BIOSS), University of Freiburg, Schänzlestr. 18, 79104 Freiburg, Germany.

*Corresponding author

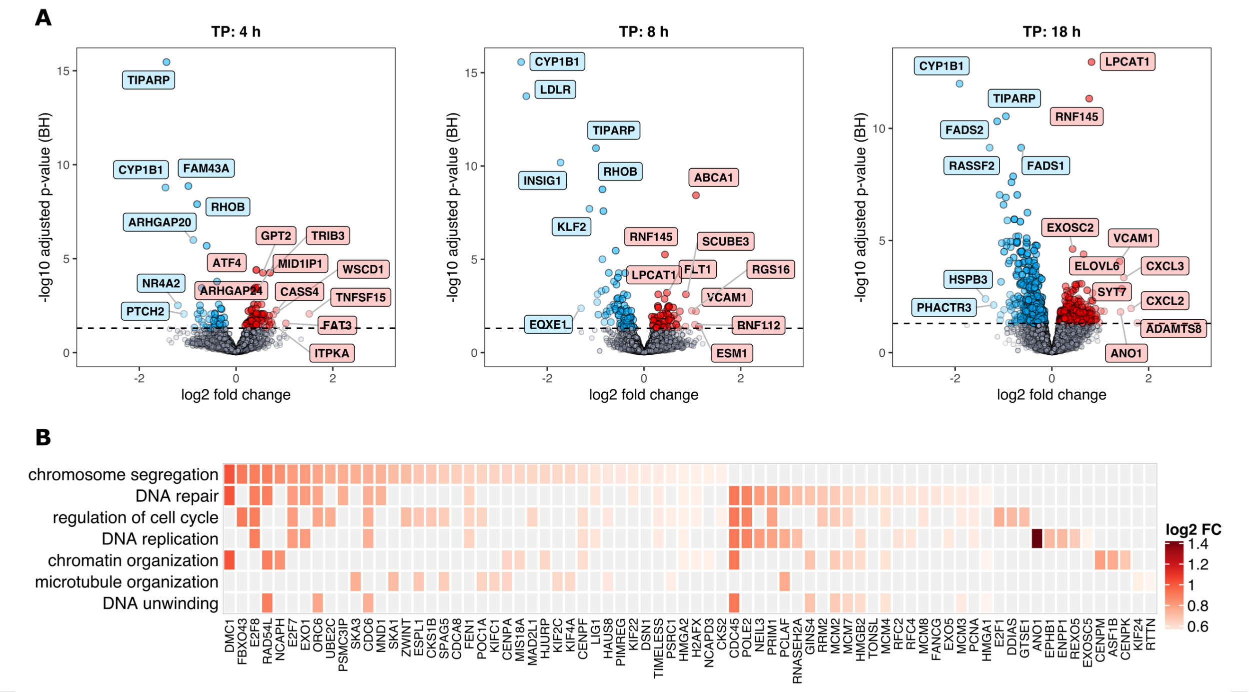

Figure S1. Differentially expressed genes in HDF after vemurafenib treatment.

(**A**) Volcano plots of 12 865 genes expressed in HDF (CPM > 1) after stimulation for different durations (TP) with 2 μM of vemurafenib. Significant up- and down-regulated genes (q < 0.05) are colored in red and blue, respectively. Average expression of the genes over all the conditions are indicated by the level of transparency of the corresponding points. The DEG with the highest significance and fold changes are labeled. (**B**) Heatmap of the most significant up-regulated genes within over-represented biological processes (q < 0.05) after 18 h of vemurafenib treatment.

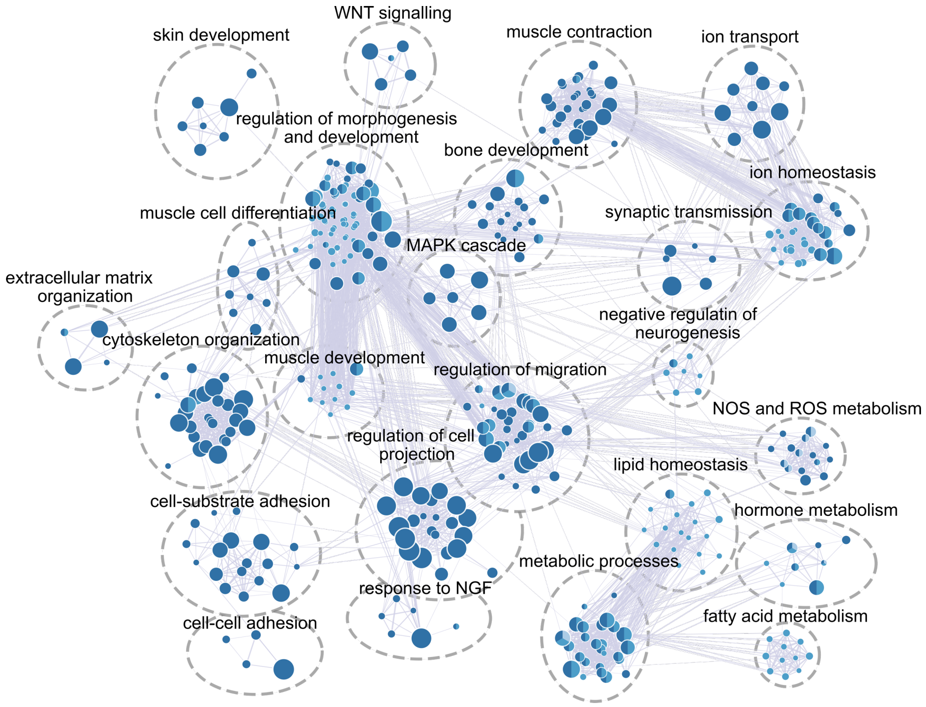

Figure S2. Down-regulated processes in HDF by vemurafenib treatment.

The plot shows the significantly over-represented ($q < 0.1$) biological processes among the genes down-regulated by treatment with 2 μM of vemurafenib. Every node represents a single GO term colored by stimulation time, with the node size indicating the number of up-regulated genes within each term. Nodes are connected based on their similarity score (combined Jaccard + Overlap score > 0.4).

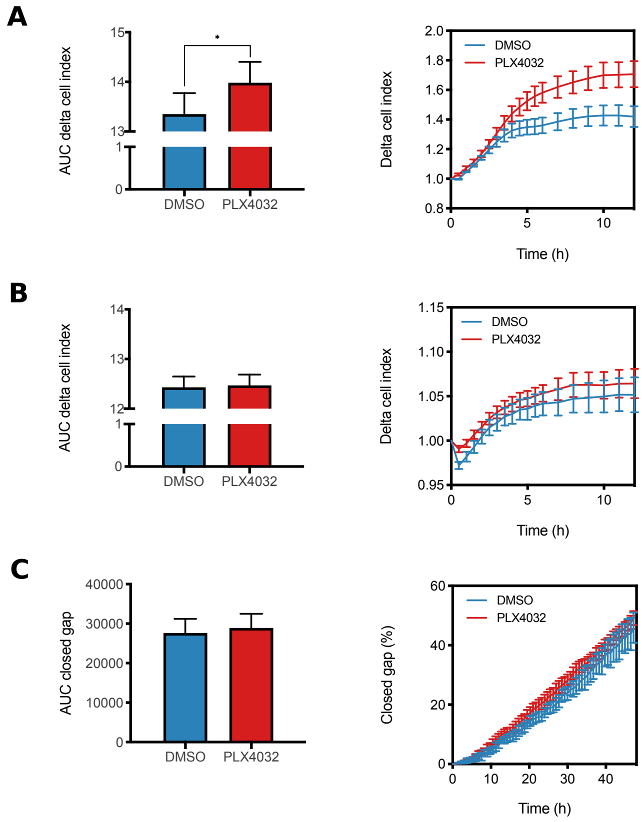

Figure S3. Effect of vemurafenib on HDF motility.

Real-time cell analysis (RTCA) of HDF (**A**) migration (chemotaxis), and (**B**) invasion, and (**C**) *in vitro* wound-healing assay, after treatment with 2 μM of vemurafenib (PLX4032) or vehicle control (DMSO). Left: the linear plots show representative curves from migrating/invading cells over time. Right: the bar plots provide the quantification of the area under the curve (AUC) for seven (migration/invasion; n = 7) or three (wound-healing; n = 3) independent experiments. All bar plots show the mean ± SEM of the experiments. *p < 0.05.

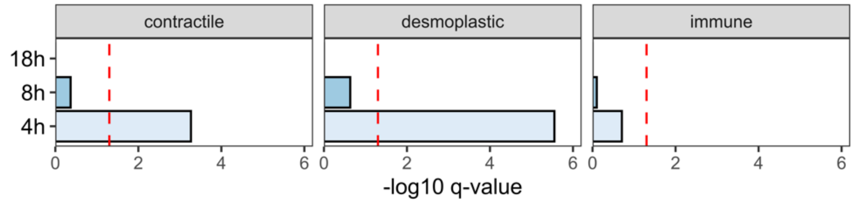

Figure S4. Enrichment of tumoral stroma signatures in HDF after vemurafenib treatment. The bar plots show the enrichment of up-regulated genes from the signatures reported by Davidson et al. (2020), for the indicated stimulation time-points. The signatures correspond to three distinct fibroblast functional-subpopulations (contractile, desmoplastic, and immune). The significance cutoff ($q < 0.05$) is shown as a dashed red line.

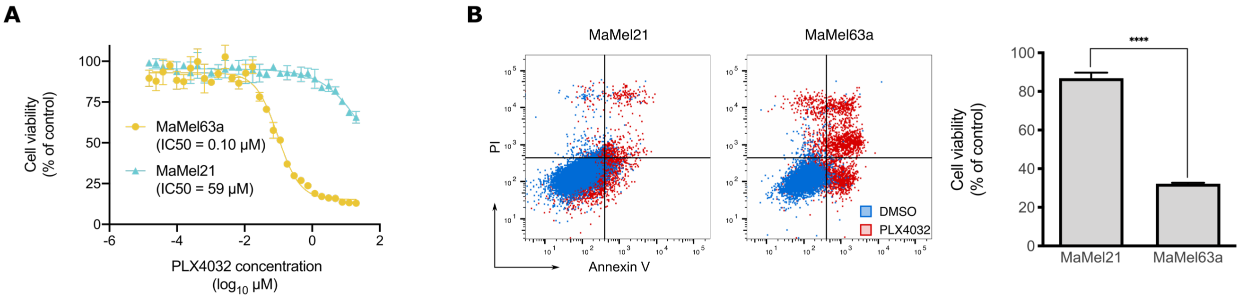

Figure S5. Effect of vemurafenib on MaMel viability.

(**A**) Cell viability of MaMel21 and MaMel63a upon treatment with vemurafenib for 48 h, as measured by MTT assay. The estimated IC50 values are indicated in parenthesis. (**B**) Flow cytometric analysis of the cell viability after 66 h of inhibition with 2 μM of vemurafenib. Left: Representative scatter plots showing the viable (lower left quadrant) and apoptotic/necrotic cells. Right: quantification of the percentage of viable cells (Annexin V – PI negative) relative to DMSO control, for three independent experiments (n = 3). The bar plot shows the mean ± SEM of the experiments. ****p < 0.0001.

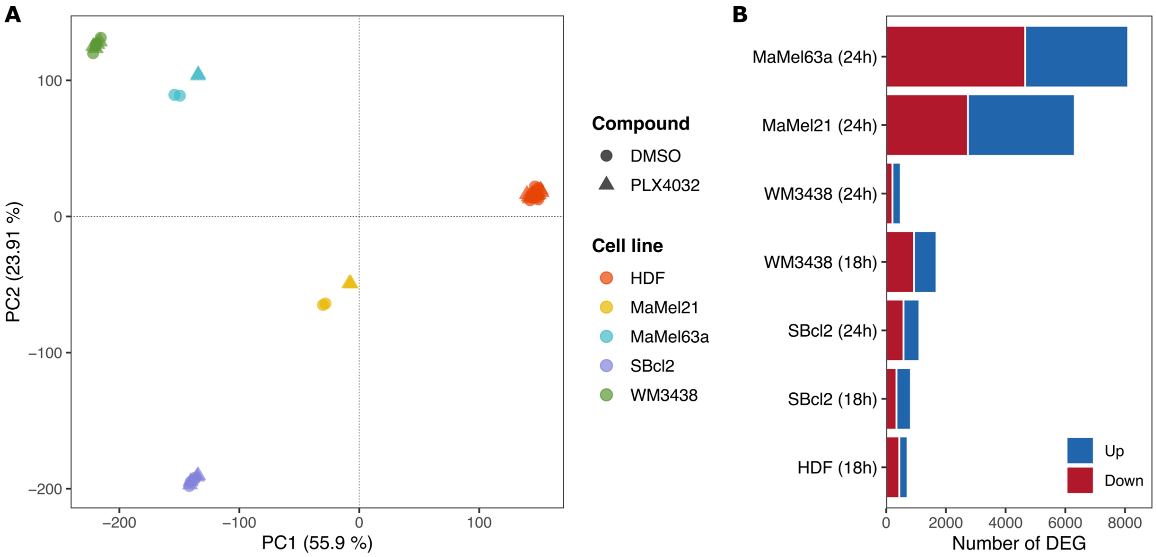

Figure S6. Comparison of transcriptional profiles in BRAF^{WT} and BRAF^{V600E} mutant cell lines after vemurafenib treatment.

BRAF^{WT} (HDF, SBcl2, and WM3438) and BRAF^{V600E} positive (MaMel21, and MaMel63a) cell lines were stimulated 2 µM of vemurafenib (PLX4032) or analog volume of vehicle (DMSO) during 18 h or 24 h. (**A**) Principal component analysis of the transcriptomics datasets. The eigenvalues from the first two main components (PC1-PC2) are plotted, and the percentage of variance explained by each one is indicated in brackets. (**B**) Bar plot showing the number of DEGs ($q < 0.05$) after vemurafenib treatment, in comparison to the corresponding vehicle-treated control. The stimulation time is indicated in parenthesis for each cell line.

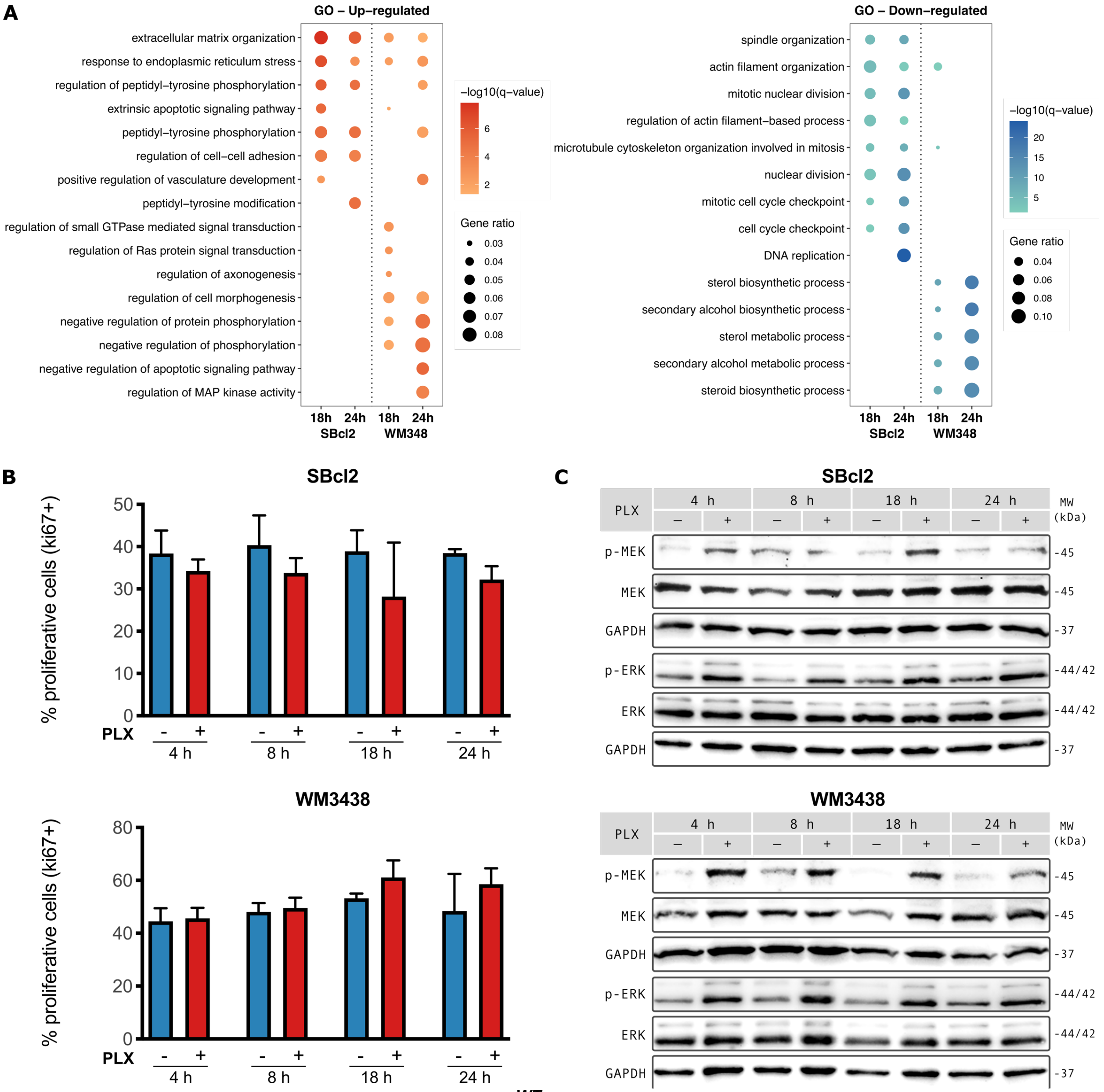

Figure S7. Effect of vemurafenib on BRAF^{WT} melanoma cell lines.

(**A**) Up- (left) and down-regulated (right) processes in SBcl2 and WM3438 cell lines after stimulation with 2 µM of vemurafenib (PLX) for the indicated time. The plots show some of the main significantly over-represented ($q < 0.05$) biological processes (GO) among the DEGs in each condition. (**B**) Flow cytometry-based determination of the proliferative state for each cell line after different stimulation times with vemurafenib. The bar plots show the quantification (mean ± SEM) of the percentage of proliferative cells (Ki67 positive) for three independent experiments (n = 3). (**C**) Western blot showing the effect of vemurafenib over MEK1/2 and ERK1/2 phosphorylation in in both cell after different stimulation times with vemurafenib. The phosphorylated (p-) and total proteins are shown. GAPDH served as a loading control.

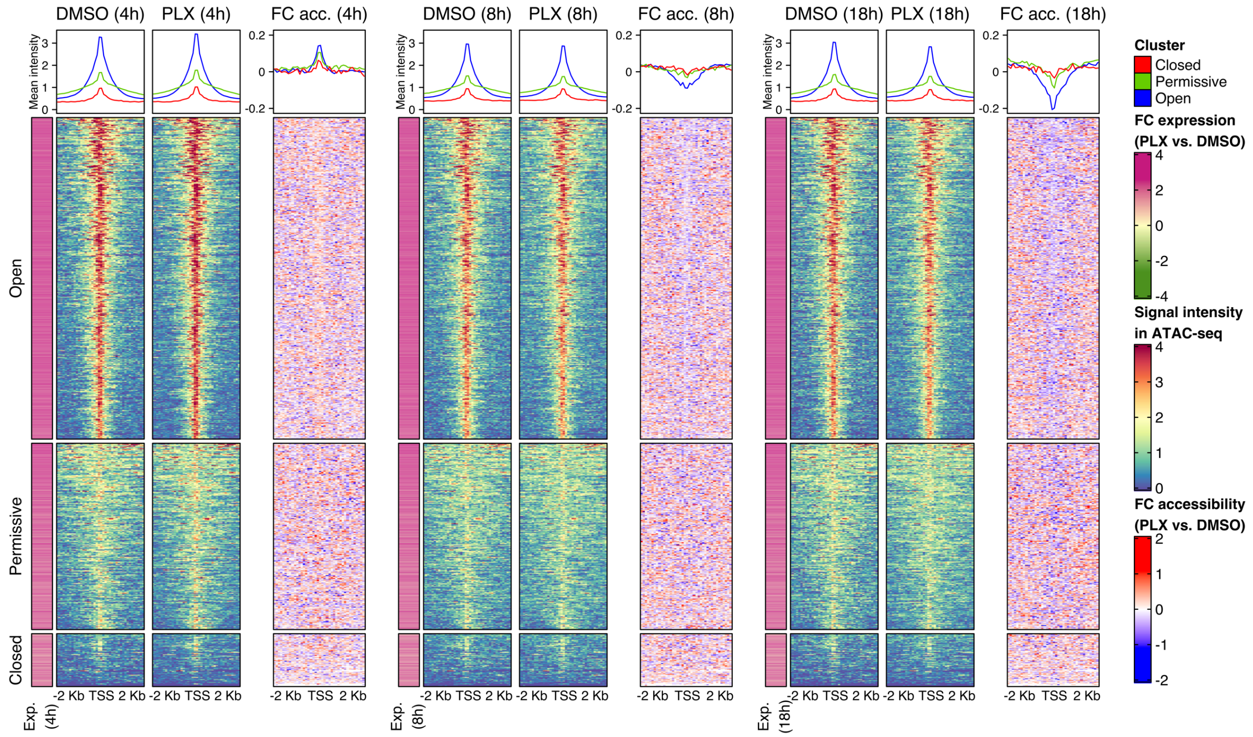

Figure S8. Accessibility within promoter regions and transcription start sites (TSS) in HDF. For each stimulation time-point (indicated in brackets) the heatmaps display the coverage of ATAC-seq reads (left, and middle), and the fold changes in accessibility (FC acc.) in vemurafenib-(PLX, for short) treated HDF, compared to DMSO control. The average gene expression (Exp.) for every stimulation time-point is represented as a left annotation. Top annotations show the mean coverage along promoter regions for each of the defined clusters (red: closed, green: permissive, blue: open).

A

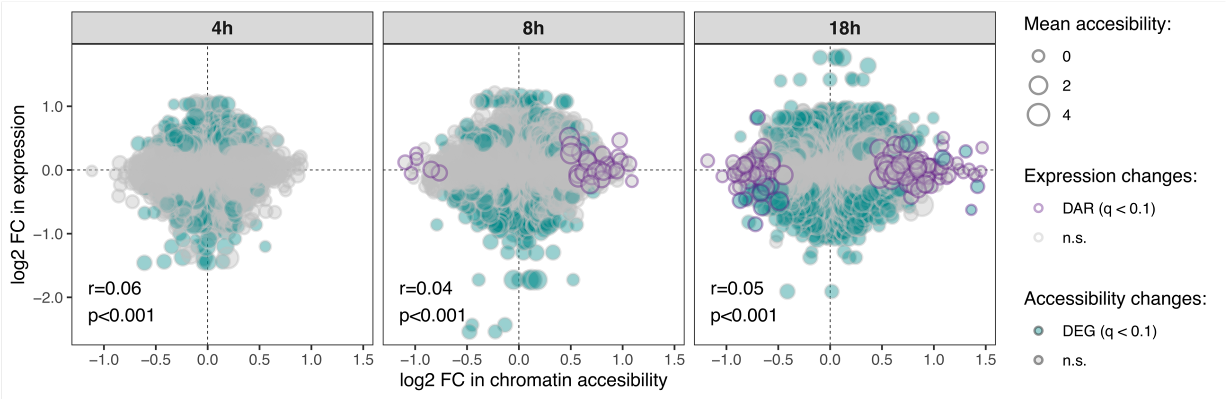

B

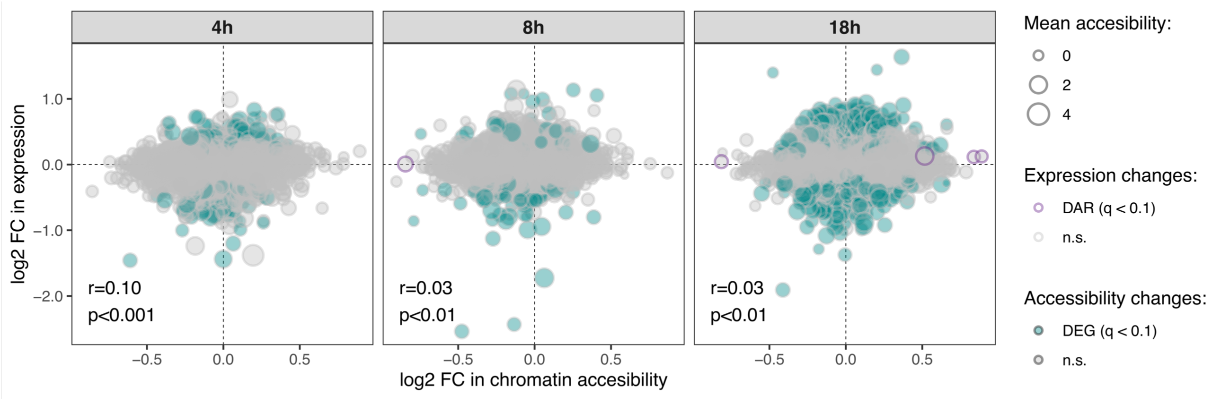

Figure S9. Pearson correlation of chromatin accessibility and gene expression changes in HDF after vemurafenib treatment.

The scatter plots show the $\log_2$ fold changes (FC) for (**A**) all the identified ATAC-seq peak regions (n = 21 800), or (**B**) promoter peaks only (n = 8062). The duration of the stimulation is indicted on top of each plot. The size of every dot represents the mean accessibility within every region. Significantly differentially accessible regions (DAR), and differentially expressed genes (DEG) are represented by the color of the stroke (purple: DAR, n.s.: non-significant), or fill (green: DEG, n.s.: non-significant), respectively.

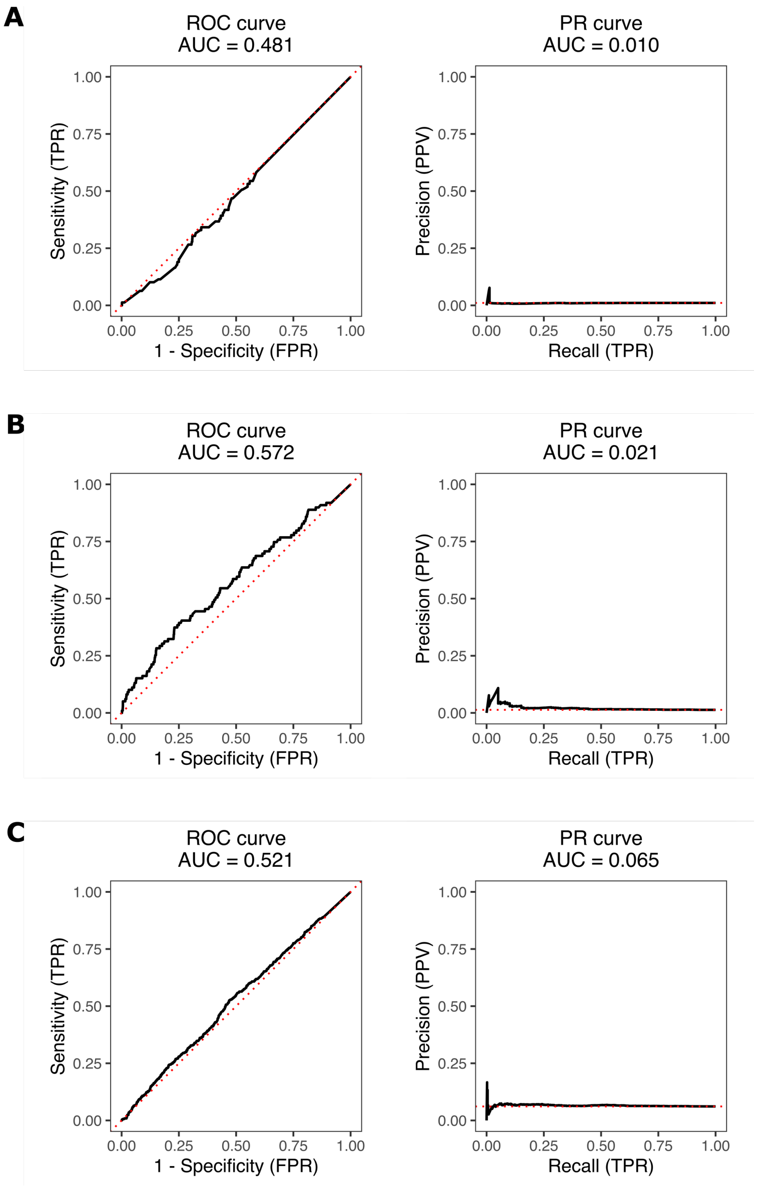

Figure S10. Receiver operating characteristic (ROC) and precision-recall (PR) curves. The curves show the potential of predicting the differentially expressed gene (DEG) status (DEG: $q < 0.05$; non-DEG: $q > 0.05$) based on the significance of accessibility changes ($-\log_{10}$ q-value) in promoter peaks, after stimulation with 2 µM of vemurafenib for (**A**) 4 h, (**B**) 8 h, or (**C**) 18 h. The area under the curve (AUC) is indicated above each plot, and the baseline is represented by the dashed red line.

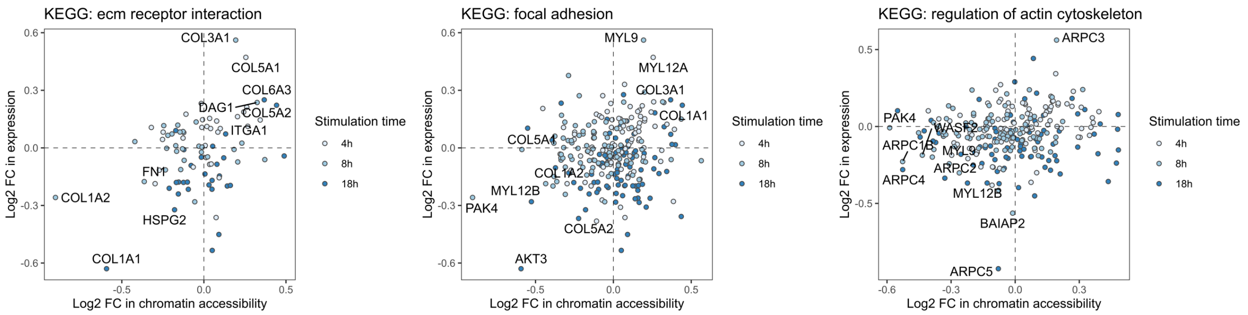

Figure S11. Most significant gene-sets (KEGG pathways) showing a relation between promoter accessibility and gene expression changes after vemurafenib treatment in HDF. Every point of the scatter plot represents a gene from the indicated pathway, during a particular stimulation time-point (indicated by the color of the point). The genes with the highest accessibility and/or expression changes are labelled.

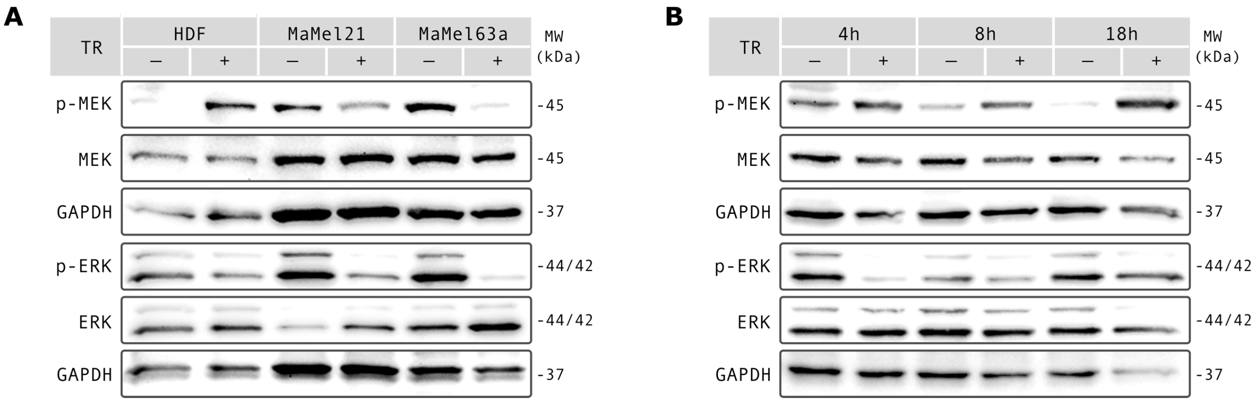

Figure S12. Effect of trametinib on MAPK/ERK pathway activation.

Representative Western blots for the phosphorylated (p-) and total MEK1/2 and ERK1/2 proteins after (**A**) 18 h-inhibition in HDF and MaMel cell, or (**B**) time-course inhibition in HDF, using 5 nM of trametinib (TR). GAPDH is shown as loading control.
